# Efficacy and Safety of PD-1/PD-L1 Checkpoint Inhibitors versus Anti-PD-1/PD-L1 Combined with Other Therapies for Tumors: A Systematic Review

**DOI:** 10.3390/cancers15030682

**Published:** 2023-01-22

**Authors:** Yiru Zhang, Qigu Yao, Yong Pan, Xinru Fang, Haoying Xu, Tingxiao Zhao, Guangqi Zhu, Tianan Jiang, Shibo Li, Hongcui Cao

**Affiliations:** 1 State Key Laboratory for the Diagnosis and Treatment of Infectious Diseases, The First Affiliated Hospital, Zhejiang University School of Medicine, 79 Qingchun Rd., Hangzhou 310003, China; 2 Department of Infectious Disease, Zhoushan Hospital, Wenzhou Medical University, 739 Dingshen Rd., Zhoushan 316021, China; 3 Department of Ultrasound Medicine, The First Affiliated Hospital, Zhejiang University School of Medicine, Hangzhou 310003, China; 4 Key Laboratory of Pulsed Power Translational Medicine of Zhejiang Province, 79 Qingchun Rd., Hangzhou 310003, China; 5 Key Laboratory of Diagnosis and Treatment of Aging and Physic-Chemical Injury Diseases of Zhejiang Province, 79 Qingchun Rd., Hangzhou 310003, China

**Keywords:** PD-1/PD-L1, combination therapy, tumors, meta-analysis

## Abstract

**Simple Summary:**

There is a wide range of choices for combining PD-1/PD-L1 checkpoint inhibitors with other measures in treating tumors, but certain choices of regimens are still controversial. We aimed to investigate the therapeutic efficacy and potential side effects of combination therapy with anti-PD-1/PD-L1 compared to anti-PD-1/PD-L1 monotherapy. Meanwhile, we also explored whether different combination strategies would yield consistent efficacy across different types of tumors. Our study can provide important and useful information for clinicians and encourage the enrollment of more clinical trials for anti-PD-1/PD-L1 combination therapy.

**Abstract:**

Objective: In recent years, the anti-programmed cell death protein-1 and its ligand (PD-1/PD-L1) or combination therapies have been recommended as an alternative emerging choice of treatment for oncology patients. However, the efficacy and adverse events of different combination strategies for the treatment of tumors remain controversial. Methods: PubMed, Embase, Cochrane Library, the American Society of Clinical Oncology (ASCO), and the European Society of Medicine Oncology (ESMO) were searched from database inception until 16 February 2022. The endpoints of objective response rate (ORR), disease control rate (DCR), overall survival (OS), progression-free survival (PFS), and adverse events (AEs) were analyzed from different treatment schemes and tumor types. The protocol was registered in PROSPERO (CRD42022328927). Results: This meta-analysis included forty-eight eligible studies. Combination therapy has improved ORR (RR = 1.40, *p* < 0.001), DCR (RR = 1.22, *p* < 0.001), and PFS (the median survival ratio (MSR) was estimated to be 1.475 *p* < 0.001) compared to anti-PD-1/PD-L1 but had no significant benefit on OS (MSR was estimated to be 1.086 *p* = 0.117). Besides, combination treatment strategies are more toxic in any grade AEs (RR = 1.13, *p* < 0.001) and grade 3–5 AEs (RR = 1.81, *p* < 0.001). Conclusions: Treatment with PD-1/PD-L1 inhibitors in combination with other antitumor therapies improve patients’ ORR, DCR, and PFS compared to anti-PD-1/PD-L1. However, it is regrettable that there is no benefit to OS and an increased risk of AEs in combinatorial therapies.

## 1. Introduction

In the new global economy, tumors have become a central issue for health. Data from a study suggest that there are approximately 19.3 million new cancer cases and 10.0 million cancer deaths in 2020 worldwide, projected to grow by more than 47% in 2040, which poses a serious threat to the quality of human life [1]. Consequently, how to choose an anti-cancer regimen has long been a question of great interest in a wide range of fields. From the discovery of the cytotoxic T lymphocyte-associated protein 4 (CTLA-4) in the 1980s to the programmed cell death protein 1 (PD-1) in the 1990s [2], immunotherapy has shown promising potential in various malignancies [3]. This shows a need to be explicit about exactly what is meant by the word PD-1. PD-1 is an immune checkpoint receptor in the tumor microenvironment (TME) [4], which is expressed on activated T cells, B cells, and natural killer cells [5]. When binding with the ligand PD-L1 or PD-L2, two motifs of PD-1 were phosphorylated, ending in the suppression of T cell proliferation and response. Therefore, blocking PD-1/PD-L1 with antibodies can strengthen pre-existent antitumor immune activity, which provides patients with a durable anti-tumor immune response [5]. Since the approval of ipilimumab in 2011, immune checkpoint inhibitors (ICIs) have been attracting a lot of interest, and seven of them for the PD-1/PD-L1 pathway have been approved by the FDA (U.S. Food and Drug Administration) so far [6]. This emerging immunomodulatory pathway was recognized by the 2018 Nobel Prize in Medicine and Physiology, which has revolutionized the traditional therapeutic approach to cancer and offers numerous options for cancer treatment [7].

Although this drug is promising, one study reported that one-third of patients had relapsed due to drug resistance [8]. Sharma has discussed the mechanisms of drug resistance, providing the theoretical basis for the combination therapy of PD-1/PD-L1 inhibitors with other treatment strategies to overcome drug resistance [9]. This is why most new trials have been combinatorial therapies since 2014, which to overcome drug resistance associated with monotherapy [10]. For example, one study by Wei examined the trend in anti-PD-1 and anti-CTLA4 combination strategies with favorable outcomes compared to monotherapy [11]. As of December 2021, 5683 clinical trials are assessing the effectiveness of anti-PD-1/PD-L1, with combination therapies leading the way (83%) and monotherapy continuing to decline (17%) [10]. However, a broad-scale study by Boyer reached different conclusions, finding that response rates remain modest for pembrolizumab plus ipilimumab [12]. It is therefore questionable whether different combination strategies and different tumor types produce consistent efficacy. Another point is that monotherapy is usually well-tolerated [13], but does the value of the efficacy brought by the current crowd-promoted combination therapy fail to balance the corresponding adverse effects in most studies? To date, these problems have received scant attention in the current meta-analysis.

Our group has previously explored the efficacy of PD-1/PD-L1 inhibitors plus other therapeutic regimens versus other therapeutic regimens and found the combination to be superior [14]. To further explore, we conducted a meta-analysis of eligible clinical trials to assess the efficacy and safety of single-agent anti-PD-1/PD-L1 versus anti-PD-1/PD-L1 combination therapy in order to provide clinical guidance on the choice of treatment options for patients with different tumors.

## 2. Materials and Methods

### 2.1. Search Strategy

We systematically scanned the associated literature published from the inception of the database to 16 February 2022 via a search of PubMed, Embase, and the Cochrane Library. There were no restrictions on region, age, gender, follow-up time, or tumor type. Combined with the retrieval strategy of Medical Subject Headings (MeSH) terms and free text words, we searched for a combination of the following terms: “PD-1”, “PD-L1”, and “tumor”. The specific search form used in PubMed can be downloaded in Supplementary Table S1. In addition to this, we have browsed American Society of Clinical Oncology (ASCO), clinicaltrials.gov, and European Society of Medicine Oncology (ESMO) for related resources that may be useful.

### 2.2. Selection Criteria

Studies were considered if they met the inclusion criteria as follows: (a) prospective clinical studies and the clinical registration number is available; (b) patients diagnosed with tumor received a combination of PD-1/PD-L1 inhibitors and other therapies (immunotherapy, targeted therapy, radiotherapy, chemotherapy, and other immune regulatory factors) in the experimental group, while treated with anti-PD-1/PD-L1 in the control group; (c) studies reported any of the following endpoints: objective response rate (ORR), disease control rate (DCR), overall survival (OS), progression-free survival (PFS), and adverse events (AEs).

The following studies have been excluded to minimize the risk of bias: (a) not in English; (b) editorials, comments, and case reports; (c) basic experiments; (d) multiple articles that analyzed the same trials.

### 2.3. Data Extraction Quality Assessment

Data were extracted independently by two researchers (Y. Zhang and Q. Yao) whose disagreements would be settled to reach a majority decision by a third investigator (H. Cao). The following information was picked up from forty-eight studies: first author, publication year, study phase, tumor type, sample size, interventions, PubMed Unique Identifier (PMID), registration number, median follow-up, median age, region, gender, primary results (containing ORR, DCR, median overall survival (mOS), the hazard ratio of OS, median progression-free survival (mPFS), the hazard ratio of PFS), and secondary AEs events (involving any-grade AEs and grade 3–5 AEs). This meta-analysis is composed according to the guidance of Preferred Reporting Items for Systematic Reviews and Meta-Analyses (PRISMA) 2020.

### 2.4. Statistical Analysis

The risk ratio (RR) of ORR, DCR, and AEs and the hazard ratio (HR) of OS and PFS were calculated using Review Manager 5.4 (https://training.cochrane.org/, accessed on 16 April 2022). Due to the small number of articles providing HR directly, we also computed summary estimates for mOS and mPFS using Stata Statistical Software 16 (Stata Corp., College Station, TX, USA). In addition to the subgroup analysis of different tumor types and treatment schemes, we also used Cochran’s Q chi-square test and *I*^2^ statistics to evaluate the heterogeneity across studies (*p* < 0.1 and *I*^2^ > 50% were considered highly heterogeneous and a random-effects model was chosen, and vice versa for a fixed-effects model). Begg’s, Egger’s, and Harbord’s tests were used when necessary to assess publication bias, with significance set at *p* < 0.1. All statistical tests were two-sided, with *p* < 0.05 being regarded as significant.

## 3. Results

### 3.1. Eligible Studies and Quality

**Figure 1:**
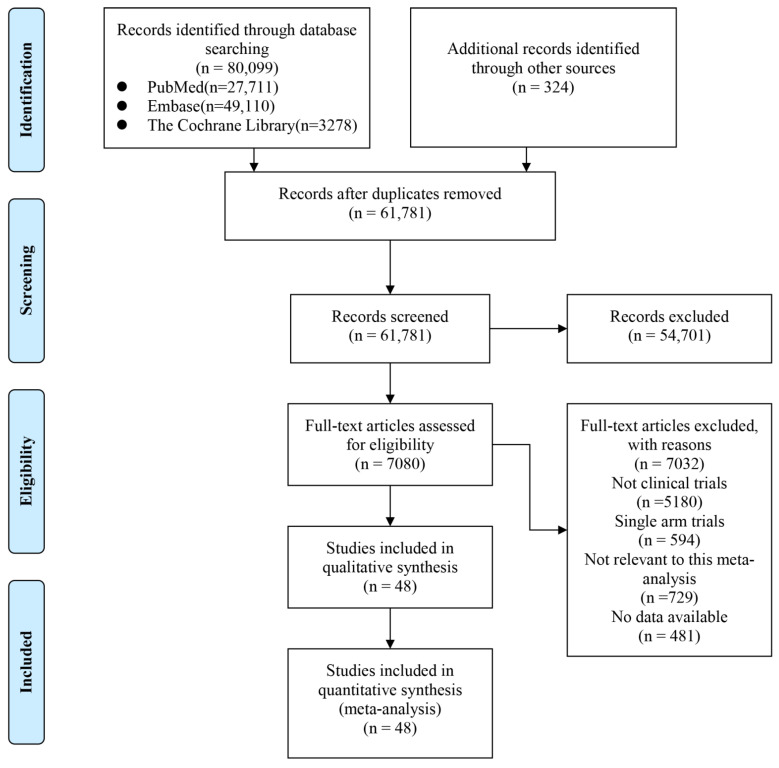
Flow chart of study selection.

Through our retrieval strategy, 80,099 articles were finally retrieved from the database. After deleting duplicates, browsing titles and abstracts, and reviewing the full text, a total of 48 eligible clinical controlled trials involving 11,385 patients were included in the meta-analysis. The detailed literature screening and selection process is illustrated in Figure 1. Furthermore, the Cochrane Collaboration tool was applied to assess the quality of the included RCT studies. The results of each project evaluated were classified as high-risk, low-risk, or unclear (Supplementary Figures S1 and S2).

### 3.2. Study Characteristics

The results demonstrate the basic characteristics of qualified research established from the database in Table 1 and Supplementary Table S2. Five treatments were covered in 48 studies, involving 26 PD-1/PD-L1 + immunotherapy vs. PD-1/PD-L1, 8 PD-1/PD-L1 + targeted therapy vs. PD-1/PD-L1, 8 PD-1/PD-L1 + chemotherapy vs. PD-1/PD-L1, 4 PD-1/PD-L1 + radiotherapy vs. PD-1/PD-L1 and 2 PD-1/PD-L1 + other drugs vs. PD-1/PD-L1. In addition, to make the results more clinically significant, we also diversified the analysis of the efficacy from the perspective of tumor type. Among the forty-eight studies, the vast majority of tumor types included digestive system tumors (*n* = 11), genitourinary system tumors (*n* = 8), non-small cell lung cancer (*n* = 8), head and neck squamous cell carcinoma (*n* = 7), and melanoma (*n* = 6). The remaining solid tumors were glioblastoma (*n* = 2), nasopharyngeal carcinoma (*n* = 1, including other pathological types such as undifferentiated in addition to squamous cell carcinoma), small cell lung cancer (*n* = 1), malignant pleural mesothelioma (*n* = 1), metastatic sarcoma (*n* = 1), and multiple tumor types (*n* =1), as well as Hodgkin’s lymphoma (*n* = 1), among hematologic system tumors.

### 3.3. Objective Response Rate (ORR)

**Figure 2:**
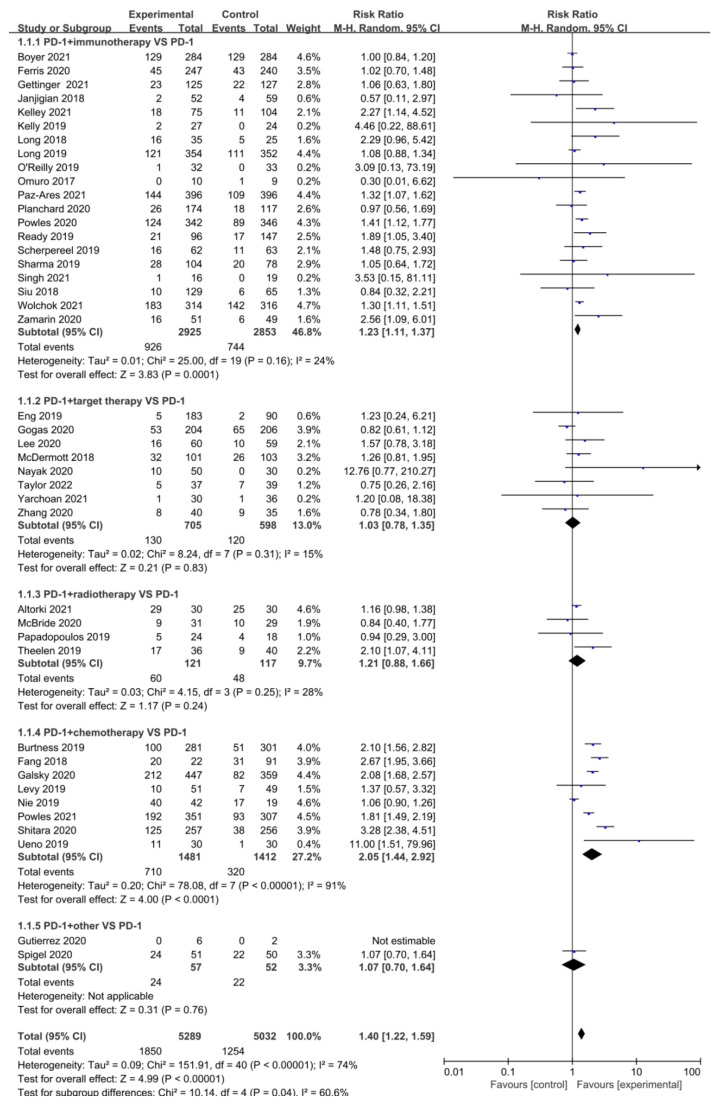
Forest Plot of the Risk ratio of ORR based on therapeutic schedules: anti-PD-1/PD-L1 versus combination therapy (immunotherapy, targeted therapy, radiotherapy, chemotherapy, and other drugs). Boyer 2021 [12], Ferris 2020 [17], Gettinger 2021 [18], Janjigian 2018 [19], Kelley 2021 [21], Kelly 2019 [22], Long 2018 [23], Long 2019 [24], Omuro 2017 [25], O’Reilly 2019 [26], Paz-Ares 2021 [27], Planchard 2020 [28], Powles 2020 [29], Ready 2019 [30], Scherpereel 2019 [31], Sharma 2019 [33], Singh 2021 [34], Siu 2018 [35], Wolchok 2021 [37], Zamarin 2020 [38], Eng 2019 [40], Gogas 2020 [41], Lee 2020 [42], McDermott 2018 [43], Nayak 2020 [44], Taylor 2022 [45], Yarchoan 2021 [46], Zhang 2020 [47], Altorki 2021 [48], McBride 2020 [49], Papadopoulos 2019 [50], Theelen 2019 [51], Burtness 2019 [52], Fang 2018 [53], Galsky 2020 [54], Levy 2019 [55], Nie 2019 [56], Powles 2021 [57], Shitara 2020 [58], Ueno 2019 [59], Gutierrez 2020 [60], Spigel 2020 [61].

The objective response rate is defined as the proportion of patients whose tumor volume shrinks to a pre-specified value and can maintain the minimum time requirement, as the sum of the proportion with complete and partial response. According to the treatment protocol, 41 of 48 studies provided direct or indirect access to ORR data. Heterogeneity tests showed greater heterogeneity in the included studies (*p* < 0.001, *I*^2^ = 74%), and effect sizes were combined using a random-effects model. In Figure 2, the results indicate that RR = 1.40, 95% CI (1.22, 1.59), and the difference in ORR was statistically significant in the experimental group compared to the control group (*p* < 0.001). PD-1/PD-L1 combined with chemotherapy and immunotherapy improved response rates of cancer clinical treatment as evidenced in subgroup analysis. In the subgroup analysis based on specific tumor types (Supplementary Figure S3), ORR was better in the combination group for NSCLC (*p* = 0.01), SCLC (*p* = 0.03), genitourinary tumors (*p* < 0.001), digestive tumors (*p* < 0.001), and nasopharyngeal carcinoma (*p* < 0.001). Among them, nasopharyngeal carcinoma and digestive tumors gained more clinical benefits, with RR = 2.67, 95% CI (1.95, 3.66) and RR = 2.41, 95% CI (1.68, 3.46), respectively.

### 3.4. Disease Control Rate (DCR)

**Figure 3:**
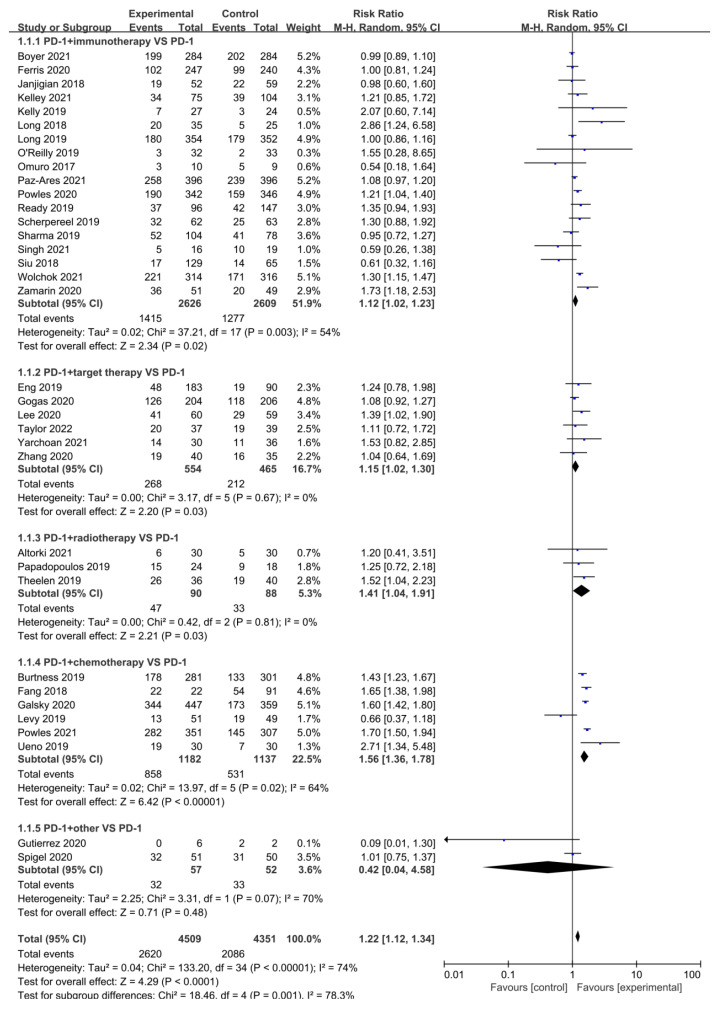
Forest Plot of the Risk ratio of DCR based on therapeutic schedules: anti-PD-1/PD-L1 versus combination therapy (immunotherapy, targeted therapy, radiotherapy, chemotherapy, and other drugs). Boyer 2021 [12], Ferris 2020 [17], Janjigian 2018 [19], Kelley 2021 [21], Kelly 2019 [22], Long 2018 [23], Long 2019 [24], Omuro 2017 [25], O’Reilly 2019 [26], Paz-Ares 2021 [27], Powles 2020 [29], Ready 2019 [30], Scherpereel 2019 [31], Sharma 2019 [33], Singh 2021 [34], Siu 2018 [35], Wolchok 2021 [37], Zamarin 2020 [38], Eng 2019 [40], Gogas 2020 [41], Lee 2020 [42], Taylor 2022 [45], Yarchoan 2021 [46], Zhang 2020 [47], Altorki 2021 [48], Papadopoulos 2019 [50], Theelen 2019 [51], Burtness 2019 [52], Fang 2018 [53], Galsky 2020 [54], Levy 2019 [55], Powles 2021 [57], Ueno 2019 [59], Gutierrez 2020 [60], Spigel 2020 [61].

Disease control rate was characterized as the proportion of patients with complete response, partial response, and stable disease among all patients. DCR was reported in 34 studies (Figure 3). Heterogeneity test: *p* < 0.001, *I*^2^ = 74%. The meta-analysis demonstrated RR = 1.22, 95% CI (1.12, 1.34), with a statistically considerable difference in the combination arm as compared to the mono-drug group (*p* < 0.001). Among the five different combination regimens, those with improved remission rates were chemotherapy [RR = 1.56, 95% CI (1.36, 1.78), *p* < 0.001], radiotherapy [RR = 1.41, 95% CI (1.04, 1.91), *p* = 0.03], and targeted therapy [RR = 1.15, 95% CI (1.02, 1.30), *p* = 0.03]. Similarly, as seen in the forest plot in Supplementary Figure S4, nasopharyngeal cancer [RR = 1.65, 95% CI (1.38, 1.98), *p* < 0.001], genitourinary tumors [RR = 1.35, 95% CI (1.11, 1.64), *p* = 0.002], and digestive system tumors [RR = 1.30, 95% CI (1.06, 1.59), *p* = 0.01] achieved varying degrees of benefit.

### 3.5. Overall Survival (OS)

**Figure 4:**
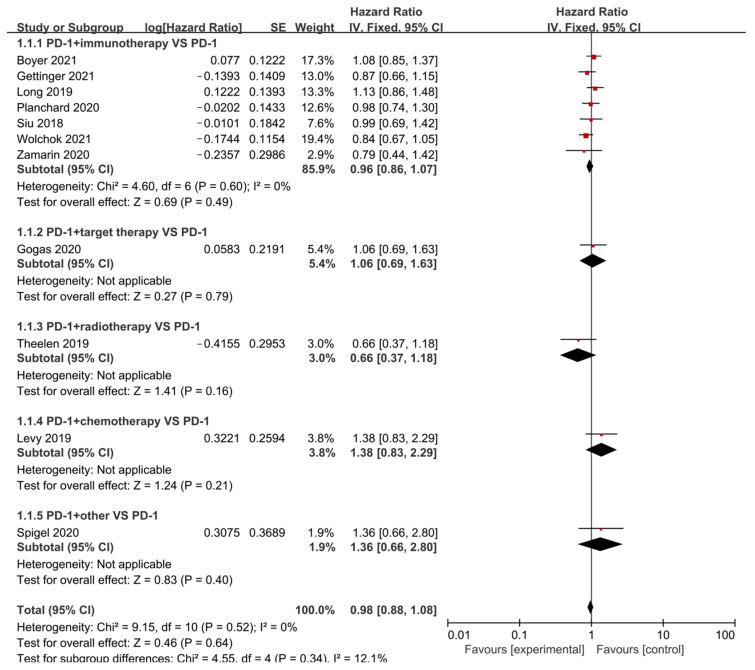
Forest Plot of the Hazard ratio of OS based on therapeutic schedules: anti-PD-1/PD-L1 versus combination therapy (immunotherapy, targeted therapy, radiotherapy, chemotherapy, and other drugs). Boyer 2021 [12], Gettinger 2021 [18], Long 2019 [24], Planchard 2020 [28], Siu 2018 [35], Wolchok 2021 [37], Zamarin 2020 [38], Gogas 2020 [41], Theelen 2019 [51], Levy 2019 [55], Spigel 2020 [61].

Overall survival was defined as the time from when the patient received treatment to the time of death from any cause or the cut-off time for follow-up. Of all the included studies, 11 articles directly provided a hazard ratio for OS. As illustrated in Figure 4 and Supplementary Figure S5, no improvement in OS in the experimental group was seen compared to the control arm (*p* = 0.64), whether analyzed by treatment or by the tumor as a subgroup.

Since the number of studies with directly available HR was not large, we further extracted the median overall survival of the articles and combined their effect sizes for a more comprehensive analysis of OS (Table 2 and Supplementary Table S3). On the basis of this analysis of overall survival time, in which there was significant heterogeneity (*I*^2^ = Please use the update Figure 4.95.2%), the median survival ratio (MSR) was estimated to be 1.086 (*p* = 0.117), indicating that the median survival time of the intervention group was 1.086 times that of the control group, and the difference was not found to be statistically significant as well. Subgroups were analyzed for differences between groups for different treatment schedules and tumor types, respectively, and their results revealed that (a) there were no statistically significant differences between the experimental group compared with the control arm for different combination regimens (*p* = 0.343), and (b) there was a statistically significant difference among the different tumor types in the combination group compared to anti-PD-1/PD-L1 (*p* < 0.001). Surprisingly, adding another drug to the treatment strategy does not appear to result in longer overall survival for tumor patients compared to anti-PD-1/PD-L1.

### 3.6. Progression-Free Survival (PFS)

Progression-free survival was defined as the time to tumor progression or follow-up endpoint after treatment. The hazard ratio of PFS was available in 13 studies. The subgroup analyses are summarized in Supplementary Figures S6 and S7. When the 13 studies were grouped by therapeutic schedules or tumor types, our meta-analysis showed that no statistically significant amelioration in PFS was observed in all groups except genitourinary tumors compared to the control group; in other words, there was no statistically significant difference between the intervention and control groups (*p* = 0.15).

Consequently, the same was applied to make full use of the data on the median progression-free survival (Table 3 and Supplementary Table S4). The number of included studies by treatment and by tumor was 38 and 37 respectively. The two numbers are different because one article describing various solid tumors did not provide a tumor-specific median PFS when classified by the tumor. As seen in both tables, there was obvious heterogeneity in the study (*I*^2^ =97.5%). In the treatment scheme, the MSR was estimated to be 1.475 (*p* < 0.001), while it was estimated to be 1.473 (*p* < 0.001) in the tumor type. The corresponding findings of this meta-analysis demonstrated that the median survival time of the intervention group was 1.475 times and 1.473 times longer than in the control arm, and there tended to be a statistical difference between the two groups as well (*p* < 0.05). It suggested that although there was no statistical difference in the risk ratio of PFS, the MSR of median progression-free survival in the experimental group was superior to that in the control group.

### 3.7. Incidence of All-Grade and Grade 3–5 Adverse Events (AEs)

A total of 48 studies with 5993 patients in the experimental groups and 5507 patients in the PD-1/PD-L1 inhibitors arm were included, and the occurrence of all-grade and grade 3–5 AEs were analyzed. Because different studies focus on a variety of AEs, we merged and selected several events with a large number of reports to analyze (Table 4 and Table 5). An analysis of the results showed that the 22 selected all-grade AEs were statistically significant in the experimental group in comparison to the control group (*p* < 0.05). However, among the six corresponding grade 3–5 AEs, RR was not statistically different (pruritus, thyroid abnormalities, pain, hypothyroidism, constipation, infection). Patients receiving combination therapy were at an increased risk of developing any grade AEs [RR = 1.13, 95% CI (1.08–1.18), *p* < 0.001] and high-grade AEs [RR = 1.81, 95% CI (1.63–2.01), *p* < 0.001] versus the monotherapy group. In our included studies, the first three most common all-grade and grade 3–5 adverse reactions were the same, all in the order of fatigue (RR = 1.31 and 1.93), diarrhea (RR = 1.87 and 3.35), and rash (RR = 1.81 and 2.07). The highest risk-averse events were both reduced platelet count, with [RR = 2.83, 95%CI (1.27, 6.29)] and [RR = 6.12, 95%CI (1.86, 20.16)] for all-grade and grade 3–5 AEs, respectively.

### 3.8. Publication Bias

Both Begg’s test and Egger’s test showed that the included studies had no significant publication bias (*p* > 0.1) in all the outcome indicators, and the Galbraith diagram (*p* > 0.1) was also drawn in ORR and DCR (Supplementary Figures S8–S11).

## 4. Discussion

With the rapid development of oncology and immunology, tumor immunotherapy has made major progress and has become another important pillar of anti-tumor treatments apart from surgery, radiotherapy, and chemotherapy, changing the therapeutic pattern of many tumors. For example, the previous treatment agent for liver cancers mainly constitutes targeted drugs and chemoradiotherapy drugs, supplemented by symptomatic drugs [62]. Now, the clinical trials of PD-1/PD-L1 inhibitors are widely carried out in various solid tumors and have been recommended for early use.

Collectively, the 48 studies in this meta-analysis outline a critical role in the efficacy and safety of PD-1/PD-L1 inhibitors in tumors. Based on the available clinical trials, which were reviewed and summarized as comprehensively as possible, there are two basic taxonomies currently being adopted in research into them in this study. One is the treatment scheme, and the other is the tumor type. The vast majority of patients experienced adverse events to varying degrees, with fatigue being particularly common. Both regimens had their pros and cons, with combination therapy showing superiority over monotherapy in ORR, DCR, and PFS, while monotherapy was superior to combination in AE. Neither was clinically significant in OS. Taken together, when making clinical treatment decisions for patients with different tumors, it may be preferable to conservatively recommend combinatorial therapies to improve patient prognosis while also considering patient tolerance to adverse effects.

The four major endpoints of oncology treatment include ORR, DCR, OS, and PFS, with the first two reflecting the near-term efficacy and the last two being the long-term efficacy indicators. The near-term efficacy indicators mainly assess the direct impact of drugs on tumors, with ORR being the most critical, while the long-term efficacy indicators mainly assess the effects of drugs on patients’ survival time, with OS being the most reliable clinical trial endpoint for evaluating antineoplastic drugs so far [63]. Therefore, we have endeavored to extract and analyze the possible clinical benefits of these four indicators for applications. A comparison of the findings with the study reported elsewhere confirms combination therapy showed numerically higher ORR [64] compared with monotherapy, but not improved OS significantly [65]. In contrast to these earlier findings, however, no evidence of the benefit of PFS was detected in this meta-analysis. The reason for this discrepancy may be attributed to the small number of studies included in this meta-analysis that directly provided the HR of OS and PFS. Since the statistical values of ORR, DCR, and PFS in the combined treatment group were improved than those of the control group, the mortality was also higher due to the higher AE. Moreover, our meta-analysis shows that compared with the control group, the combined treatment group has no advantage in OS HR. But the efficacy of clinical trials is also affected by many factors, such as accessibility and financial burden. Therefore, we propose the following solutions. First of all, symptomatic treatment of AE and prevention of 3–5 levels of AE should be carried out to reduce the death rate caused by AE. Second, more clinical studies and design trials should be included to reduce the impact of other non-research factors.

Therefore, to make the findings more convincing, we also considered median survival as a combined effect size and calculated a median survival ratio point estimate as a further argument for the above speculation. One unanticipated finding was that these results indeed further support the benefit of PFS. These findings are consistent with that of Yang (2020) [66], who also has demonstrated that combination therapy significantly improved ORR and DCR and prolonged mPFS but has not yet impacted overall survival. Inspired by the conjecture presented in the previous article, we venture to speculate that the activity of combination therapy for tumors may be more of a cumulative effect than a synergistic one.

Based on the subgroup analysis of tumor types, the overall efficacy evaluation of long- and short-term efficacy for different cancer types in combination therapy and monotherapy showed consistency with the treatment regimens. The most obvious finding to emerge from the analysis is that compared with the single-drug group, ORR, DCR, and PFS of the digestive system and genitourinary system tumors in the combined treatment group represent better clinical advantages. We observed that these two tumor types accounted for a higher percentage of the included studies. Therefore, based on these data, we infer that this result may be due to the number of studies. To go a step further, a large proportion of studies with small sample sizes are more prone to findings that contradict the conclusions drawn from the results of the combined effects. Recent systematic literature reviews concluded that the superiority of combined anti-CTLA-4 and anti-PD-1 therapy for melanoma and NSCLC in the response rates or OS has been proven to date, which is contrary to our conclusion (*p* = 0.32 and *p* = 0.1) [67,68]. In recent years, clinical studies on reversing drug resistance to anti-PD-1 by modulating intestinal flora are in full swing. In one well-known recent experiment, Baruch et al. found a significant increase in the number of immune cell infiltration in melanoma patients who had undergone fecal transplantation, successfully activating the immune response in the patients [69]. Previously, Janney et al. described various possible mechanisms by which intestinal flora affects the treatment of colorectal cancer, suggesting that modulating intestinal flora to overcome immunotherapy resistance may be another viable avenue [70]. Therefore, the other speculation is that combination therapy may be more advantageous in digestive system tumors than in tumors of other systemic origins.

The National Cancer Institute (NCI) provides a grading (severity) scale for each AE term, which allows recognition of relevant clinical manifestations for the early assessment of toxic reactions. Comparison of the findings with those of other studies [13,71,72,73,74,75] confirms that patients with combination therapy are more likely to experience AEs than those in the anti-PD-1/PD-L1 arm. The higher risk of various adverse reactions comes from the digestive system and the hematological system, suggesting that front-line clinical workers are expected to be aware at an early stage and take preventive measures as soon as possible. A recent meta-analysis mentioned that anti-PD-L1 therapy can achieve long-term tumor control by maintaining immune activation [76]. However, excessive immune activation can induce immune-related adverse events (irAE). ICIs may contribute to the development of irAEs by affecting peripheral tolerance to autoantigens, leading to the formation of autoantibodies. The association between the development of immune-related adverse events and improvement in disease prognosis are two sides of the same coin. The occurrence of irAE can be treated with supportive measures such as steroids and other immunosuppressive agents without discontinuation of ICI therapy. Therefore, early diagnoses of irAE, symptom monitoring, and patient education can prevent the exacerbation of AE to higher levels of toxicity.

This study adds to the growing body of research that indicates the response rates and survival of the various combination strategies are not consistently better than those of anti-PD-1/PD-L1. In addition, the efficacy of different types of tumors depends on the specific treatment regimen. Although various similar treatment therapies and tumors can be cross-referenced, specific problems should be analyzed, and no generalization should be made. Thirdly, as to the question of how to balance efficacy and adverse effects, the recommendation of this meta-analysis is that the more potent strategy is advised to improve patient prognosis without compromising patient life and quality of life. Last but not least, given that the combination of nivolumab and ipilimumab is not a cost-effective option compared to nivolumab therapy [77], patients’ financial burdens should also be taken into account when choosing an option.

It is reported that high monocyte count is a poor prognostic factor for patients with solid tumors [78,79]. The prognosis of colorectal cancer and breast cancer patients with neutrophil to lymphocyte ratio >2.5 or increased neutrophil to lymphocyte ratio after surgery was significantly lower [80,81]. In the literature we included, we found that the density of CD8-positive tumor-infiltrating lymphocytes (CD8TIL) was higher, which could improve the prognosis of patients with oral cancer [16].

When anti-tumor drugs are combined, the order of administration is usually determined by the principles of cell dynamics, interaction, and stimulation. In the literature search, we found that the clinical trial of anti-PD-1/PD-L1 combination therapies did not give a clear order of administration. Anti-PD-1/PD-L1, as new anti-tumor drugs, their mechanism of action is different from that of traditional anti-tumor drugs. Due to the different understanding of the interaction between anti-PD-1/PD-L1 and traditional chemicals, there are many differences in the order of their combined use in clinical practice.

Inflammatory factor IFN-γ, inflammatory cells, such as T lymphocytes, can induce high expression of PD-L1, which is helpful in inhibiting tumors [82,83]. Based on the above literature, it is reasonable to speculate that the use of anti-inflammatory drugs can cooperate with PD-1/PD-L1 inhibitors to suppress the tumor immune escape and synergistically enhance the therapeutic effect of PD-1/PD-L1 inhibitors. Current mainstream combination strategies include immuno-oncology (IO) therapies, targeted therapies, chemotherapies, and radiotherapies. Based on the number of new trials started in recent years [84], emerging therapies of interest for the future may lie in combinations with approved therapies such as PARP inhibitors, as well as rising IO targets and agents such as TIGIT, TGFβ/TGFR, TLRs, oncolytic viruses, and cancer vaccines.

Admittedly, the scope of this study was limited in the following terms. First, of the 48 prospective clinical trials enrolled in this study, three were not randomized controlled trials and were not included in the risk of bias assessment. One included two single-arm phase 1 trials; another was a non-comparative phase 2 trial that included two independent trials; the other was a non-randomized controlled clinical phase 1 study. However, these studies meet our nadir criteria and have some implications for our research. Besides, some of the trials studied multiple similarly eligible cohorts simultaneously. We did not find differences by comparison, so the two cohorts with larger sample sizes were selected to reduce bias. Second, the sum of the total number of men and women in some studies did not match the number of interventions. We browsed and summarized the included literature and found that this was because the baseline data was filled in with the initial number of people, while the intervention number represented the number of available people, and people may have dropped out of the study in the middle of the trial so that the numbers were not exactly the same. Third, this meta-analysis did not standardize tumor and adverse effect evaluation criteria. Although there were differences, most studies used RECIST version 1.1 and CTCAE version 4.0. Fourth, a majority of the included studies failed to explore patients with PD-1/PD-L1 expression greater than 50% and less than 50%. Previous research has established that the response rate for patients with PD-L1 TPS of ≥50% was found to be 45.2%, compared to 16.5% and 10.7% among patients with a TPS of 1–49% and <1%, respectively [85]. Although there are no available prospective data on the efficacy of ICI monotherapy versus ICI plus chemotherapy in patients with low tumor PD-L1 expression levels (TPS of <50%), it is suggested that more upcoming clinical trials could be conducted to discuss the efficacy separately according to the expression of PD-1/PD-L1 [86]. Finally, most of our analyses fell into the category of no or mild to moderate heterogeneity (*I*^2^ ≤ 75%) and used a more conservative random effects model. In addition, for treatments with greater heterogeneity (*I^2^* > 75%), we performed a subgroup analysis (i.e., by tumor type and treatment regimen). Eventually, high heterogeneity was found in the ORR of anti-PD-1/PD-L1 plus chemotherapy, DCR of the genitourinary system, HNSCC, melanoma, and PFS of anti-PD-1/PD-L1 plus targeted agents. The heterogeneity may be due to the fact that the number of articles eligible for inclusion is not very large.

## 5. Conclusions

The most obvious finding to emerge from this study is that PD-1/PD-L1 inhibitors combined with chemotherapy and immunotherapy improve ORR, and those with improved DCR were chemotherapy, radiotherapy, and targeted therapy. ORR was better in the combination group for NSCLC, SCLC, genitourinary tumors, digestive tumors, and nasopharyngeal carcinoma, while nasopharyngeal cancer, genitourinary tumors, and digestive system tumors perform better in DCR. PFS in the experimental group was superior to that in the control group. However, the addition of another drug to the treatment strategy does not appear to result in prolonged OS in tumor patients compared to anti-PD-1/PD-L1. In addition, all-grade and grade 3–5 AEs were greater in the experimental group compared to the control group.

## Figures and Tables

**Table 1:** Main characteristic of included studies.

Author	Phase	Tumor	Sample Size		Interventions		PMID	RN
					Experimental	Control		
Boyer 2021 [12]	III	NSCLC	284	284	Pembrolizumab + Ipilimumab	Pembrolizumab + Placebo	33513313	NCT03302234
D’Angelo 2018 [15]	II	Sarcoma	42	43	Nivolumab + Ipilimumab	Nivolumab	29370992	NCT02500797
Ferrarotto 2020 [16]	I	Oropharynx cancer	14	15	Durvalumab + Tremelimumab	Durvalumab	32269052	NCT03144778
Ferris 2020 [17]	III	HNSCC	247	240	Durvalumab + Tremelimumab	Durvalumab	32294530	NCT02369874
Gettinger 2021 [18]	III	NSCLC	125	127	Nivolumab + ipilimumab	Nivolumab	34264316	NCT02785952
Janjigian 2018 [19]	I/II	Esophagogastric cancer	52	59	Nivolumab + ipilimumab	Nivolumab	30110194	NCT01928394
Kaseb 2022 [20]	II	HCC	14	13	Nivolumab + ipilimumab	Nivolumab	35065057	NCT03222076
Kelley 2021 [21]	I/II	HCC	75	104	Durvalumab + tremelimumab	Durvalumab	34292792	NCT02519348
Kelly 2019 [22]	Ib/II	Gastric or GEJ cancer	27	24	Durvalumab + tremelimumab	Durvalumab	31676670	NCT02340975
Long 2018 [23]	II	Melanoma	35	25	Nivolumab + ipilimumab	Nivolumab	29602646	NCT02374242
Long 2019 [24]	III	Melanoma	354	352	Pembrolizumab + Epacadostat	Pembrolizumab + Placebo	31221619	NCT02752074
Omuro 2017 [25]	I	Mlioblastoma	10	10	Nivolumab + Ipilimumab	Nivolumab	29106665	NCT02017717
O’Reilly 2019 [26]	II	mPDAC	32	32	Durvalumab + Tremelimumab	Durvalumab	31318392	NCT02558894
Paz-Ares 2021 [27]	III	NSCLC	396	396	Nivolumab + Ipilimumab	Nivolumab	34648948	NCT02477826
Planchard 2020 [28]	III	NSCLC	174	117	Durvalumab + Tremelimumab	Durvalumab	32201234	NCT02352948
Powles 2020 [29]	III	Urothelial carcinoma	342	346	Durvalumab + Tremelimumab	Durvalumab	32971005	NCT02516241
Ready 2019 [30]	I/II	SCLC	96	147	Nivolumab + Ipilimumab	Nivolumab	31629915	NCT01928394
Scherpereel 2019 [31]	II	MPM	62	63	Nivolumab + Ipilimumab	Nivolumab	30660609	NCT02716272
Schoenfeld 2020 [32]	II	OCSCC	15	14	Nivolumab + Ipilimumab	Nivolumab	32852531	NCT02919683
Sharma 2019 [33]	I/II	Urothelial carcinoma	104	78	Nivolumab + Ipilimumab	Nivolumab	31100038	NCT01928394
Singh 2021 [34]	II	GIST	16	19	Nivolumab + Ipilimumab	Nivolumab	34407970	NCT02880020
Siu 2018 [35]	II	HNSCC	133	67	Durvalumab + Tremelimumab	Durvalumab	30383184	NCT02319044
Tawbi 2022 [36]	II/III	Melanoma	355	359	Nivolumab + Relatlimab	Nivolumab	34986285	NCT03470922
Wolchok 2021 [37]	III	Melanoma	314	316	Nivolumab + Ipilimumab	Nivolumab	34818112	NCT01844505
Zamarin 2020 [38]	II	EOC	51	49	Nivolumab + Ipilimumab	Nivolumab	32275468	NCT02498600
Zimmer 2020 [39]	II	Melanoma	56	59	Nivolumab + Ipilimumab	Nivolumab	32416781	NCT02523313
Eng 2019 [40]	III	Colorectal cancer	183	90	Atezolizumab + Cobimetinib	Atezolizumab	31003911	NCT02788279
Gogas 2020 [41]	III	Melanoma	222	224	Atezolizumab + Cobimetinib	Pembrolizumab	33309774	NCT03273153
Lee 2020 [42]	Ib	HCC	60	59	Atezolizumab + Bevacizumab	Atezolizumab	32502443	NCT02715531
McDermott 2018 [43]	II	RCC	101	103	Atezolizumab + Bevacizumab	Atezolizumab	29867230	NCT01984242
Nayak 2020 [44]	II	Glioblastoma	50	30	Pembrolizumab + Bevacizumab	Pembrolizumab	33199490	NCT02337491
Taylor 2022 [45]	II	HNSCC	37	39	Pembrolizumab + Acalabrutinib	Pembrolizumab	34862248	NCT02454179
Yarchoan 2021 [46]	II	BTC	38	39	Atezolizumab + Cobimetinib	Atezolizumab	34907910	NCT03201458
Zhang 2020 [47]	II	Urothelial carcinoma	40	35	Pembrolizumab + Acalabrutinib	Pembrolizumab	32757302	NCT02351739
Altorki 2021 [48]	II	NSCLC	30	30	Durvalumab + SBRT	Durvalumab	34015311	NCT02904954
McBride 2020 [49]	II	HNSCC	32	30	Nivolumab + SBRT	Nivolumab	32822275	NCT02684253
Papadopoulos 2019 [50]	I	Solid Tumors	24	18	Cemiplimab + hfRT	Cemiplimab	31796520	NCT02383212
Theelen 2019 [51]	II	NSCLC	36	40	Pembrolizumab + SBRT	Pembrolizumab	31294749	NCT02492568
Burtness 2019 [52]	III	HNSCC	281	301	Pembrolizumab + Chemotherapy	Pembrolizumab	31679945	NCT02358031
Fang 2018 [53]	I	NPC	23	93	Camrelizumab + Chemotherapy	Camrelizumab	30213452	NCT02721589NCT03121716
Galsky 2020 [54]	III	Urothelial carcinoma	451	362	Atezolizumab + Chemotherapy	Atezolizumab	32416780	NCT02807636
Levy 2019 [55]	II	NSCLC	51	49	Pembrolizumab + CC-486	Pembrolizumab + Placebo	30654297	NCT02546986
Nie 2019 [56]	II	cHL	42	19	Camrelizumab + Decitabine	Camrelizumab	31039052	NCT02961101NCT03250962
Powles 2021 [57]	III	Urothelial carcinoma	351	307	Pembrolizumab + Chemotherapy	Pembrolizumab	34051178	NCT02853305
Shitara 2020 [58]	III	Gastric Cancer	257	256	Pembrolizumab + Chemotherapy	Pembrolizumab	32880601	NCT02494583
Ueno 2019 [59]	I	BTC	30	30	Nivolumab + Chemotherapy	Nivolumab	31109808	JapicCTI-153098
Gutierrez 2020 [60]	I/IIa	Bladder cancer	6	2	BMS-986178 + Nivolumab	Nivolumab	33148673	NCT02737475
Spigel 2020 [61]	II	NSCLC	51	50	Pembrolizumab + Pegilodecakin	Pembrolizumab	33166722	NCT03382899NCT03382912

Abbreviations: NSCLC, non-small cell lung cancer; HNSCC, head and neck squamous cell carcinoma; HCC, hepatocellular carcinoma; GEJ, gastroesophageal junction; mPDAC, metastatic pancreatic ductal adenocarcinoma; SCLC, small cell lung cancer; MPM, malignant pleural mesothelioma; OCSCC, Oral Cavity Squamous Cell Carcinoma; GIST, Gastrointestinal Stromal Tumors; EOC, epithelial ovarian cancer; RCC, renal cell carcinoma; BTC, biliary tract cancer; NPC, nasopharyngeal carcinoma; cHL, classic Hodgkin lymphoma; SBRT, stereotactic body radiotherapy; hfRT, hypofractionated radiotherapy, PMID, PubMed Unique Identifier; RN, Registration Number. Three studies had the same NCT number because the clinical trial investigated six tumor types simultaneously and published multiple articles with the same number (NCT01928394). One study analyzed two single-arm phase 1 trials of patients with nasopharyngeal carcinoma and had the same primary outcome indicators, but was a non-randomized controlled trial (NCT02721589, NCT03121716). Both clinical trials enrolled in this single-center study investigated anti-PD-L1 alone or in combination with decitabine for Hodgkin’s lymphoma, with essentially identical inclusion and exclusion criteria and the same primary endpoint (NCT02961101, NCT03250962). The study included two NCT numbers (NCT03382899, NCT03382912) because Pegilodecakin combined two kinds of PD-L1. Since both cohorts had the same outcome, we selected the larger cohort for statistical purposes (NCT03382899).

**Table 2:** Median survival ratio of OS based on therapeutic schedules.

Subgroup and Author (Year)	ES	[95% Conf. Interval]		%Weight
PD-1 + Immunotherapy vs. PD-1				
Omuro 2017 [25]	0.885	0.564	1.387	2.17
Schrpereel 2019 [31]	1.336	1.121	1.592	3.35
Singh 2021 [34]	0.327	0.235	0.456	2.68
Zamarin 2020 [38]	1.289	1.060	1.568	3.27
Planchard 2020 [28]	1.150	1.025	1.290	3.55
O’Reilly 2019 [26]	0.861	0.675	1.098	3.08
Wolchok 2021 [37]	1.954	1.807	2.113	3.64
Siu 2018 [35]	1.267	1.100	1.458	3.48
Paz-Ares 2021 [27]	1.089	1.016	1.168	3.66
Boyer 2021 [12]	0.977	0.900	1.061	3.63
Ready 2019 [30]	0.825	0.727	0.935	3.52
Sharma 2019 [33]	0.747	0.646	0.864	3.46
Ferris 2020 [17]	0.855	0.783	0.935	3.62
Kelley 2021 [21]	1.238	1.070	1.434	3.46
Kelly 2019 [22]	2.706	2.056	3.560	2.94
D’Angelo 2018 [15]	1.336	1.081	1.653	3.21
Gettinger 2021 [18]	0.909	0.804	1.029	3.53
Powles 2020 [29]	1.144	1.062	1.233	3.65
Janjigian 2018 [19]	0.774	0.643	0.932	3.31
Subgroup, DL	1.059	0.920	1.220	63.20
PD-1 + target therapy vs. PD-1				
Eng 2019 [40]	1.249	1.110	1.407	3.54
Nayak 2020 [44]	0.854	0.686	1.064	3.18
Taylor 2022 [45]	1.010	0.807	1.265	3.16
Zhang 2020 [47]	0.553	0.441	0.693	3.15
Subgroup, DL	0.885	0.620	1.262	13.03
PD-1 + radiotherapy vs. PD-1				
McBride 2020 [49]	0.979	0.760	1.261	3.03
Theelen 2019 [51]	2.092	1.671	2.620	3.16
Subgroup, DL	1.434	0.681	3.019	6.19
PD-1 + chemotherapy vs. PD-1				
Burtness 2019 [52]	0.872	0.804	0.946	3.63
Shitara 2020 [58]	1.179	1.081	1.286	3.62
Ueno 2019 [59]	2.962	2.299	3.814	3.03
Galsky 2020 [54]	1.019	0.951	1.092	3.66
Powles 2021 [57]	1.090	1.010	1.176	3.64
Subgroup, DL	1.222	1.006	1.484	17.59
Overall, DL	1.086	0.980	1.203	100.00

Tests of subgroup effect size = 1; Cochran’s Q statistics for heterogeneity; PD-1 + immunotherapy vs. PD-1: z =0.797, *p*= 0.426; *I*^2^ =95.8%; PD-1 + target therapy vs. PD-1: z =-0.676, *p* = 0.499; *I*^2^= 92.8%; PD-1 + radiotherapy vs. PD-1: z =0.950, *p* = 0.342; *I*^2^ = 94.8%; PD-1 + chemotherapy vs. PD-1: z =2.024, *p* = 0.043; *I^2^* = 95.7%; Overall: z =1.568, *p* = 0.117; *I*^2^ = 95.2%; Between: *p* = 0.343.

**Table 3:** Median survival ratio of PFS based on therapeutic schedules.

Subgroup and Author (Year)	ES	[95% Conf. Interval]		% Weight
PD-1 + Immunotherapy vs. PD-1				
Kaseb 2022 [20]	2.078	1.425	3.030	2.29
Omuro 2017 [25]	0.789	0.504	1.238	2.13
Scherpereel 2019 [31]	1.400	1.175	1.668	2.67
Singh 2021 [34]	0.710	0.510	0.989	2.39
Zamarin 2020 [38]	1.950	1.603	2.372	2.64
Planchard 2020 [28]	1.129	1.006	1.266	2.74
O’Reilly 2019 [26]	1.000	0.784	1.275	2.56
Long 2019 [24]	0.959	0.891	1.033	2.77
Long 2018 [23]	5.308	4.121	6.836	2.54
Tawbi 2022 [36]	2.196	2.040	2.363	2.77
Wolchok 2021 [37]	1.667	1.541	1.802	2.77
Siu 2018 [35]	1.053	0.914	1.212	2.71
Paz-Ares 2021 [27]	1.214	1.133	1.302	2.77
Boyer 2021 [12]	0.976	0.899	1.060	2.76
Ready 2019 [30]	1.071	0.945	1.215	2.73
Sharma 2019 [33]	0.929	0.803	1.074	2.70
Kelley 2021 [21]	1.048	0.905	1.214	2.70
Kelly 2019 [22]	1.125	0.855	1.480	2.50
D’Angelo 2018 [15]	2.412	1.950	2.983	2.61
Gettinger 2021 [18]	1.310	1.158	1.483	2.73
Powles 2020 [29]	1.609	1.493	1.734	2.77
Janjigian 2018 [19]	1.143	0.949	1.377	2.65
Subgroup, DL	1.337	1.157	1.544	57.88
PD-1 + target therapy vs. PD-1				
Eng 2019 [40]	0.985	0.874	1.109	2.73
McDermott 2018 [43]	1.918	1.672	2.200	2.71
Gogas 2020 [41]	0.965	0.876	1.063	2.75
Nayak 2020 [44]	2.867	2.303	3.570	2.60
Yarchoan 2021 [46]	1.952	1.533	2.484	2.56
Taylor 2022 [45]	1.588	1.268	1.989	2.59
Lee 2020 [42]	1.647	1.376	1.971	2.66
Zhang 2020 [47]	1.375	1.097	1.724	2.59
Subgroup, DL	1.559	1.189	2.044	21.20
PD-1 + radiotherapy vs. PD-1				
Papadopoulos 2019 [50]	1.583	1.170	2.142	2.45
McBride 2020 [49]	1.368	1.063	1.762	2.54
Theelen 2019 [51]	3.474	2.774	4.349	2.59
Subgroup, DL	1.968	1.068	3.625	7.58
PD-1 + chemotherapy vs. PD-1				
Burtness 2019 [52]	2.130	1.964	2.311	2.76
Levy 2019 [55]	0.725	0.596	0.882	2.64
Shitara 2020 [58]	3.450	3.164	3.762	2.76
Ueno 2019 [59]	3.000	2.329	3.864	2.54
Subgroup, DL	2.004	1.178	3.408	10.70
PD-1 + other vs. PD-1				
Spigel 2020 [61]	1.033	0.850	1.255	2.64
Subgroup, DL	1.033	0.850	1.255	2.64
Overall, DL	1.475	1.290	1.688	100.00

Tests of subgroup effect size = 1; Cochran’s Q statistics for heterogeneity. PD-1 + immunotherapy vs. PD-1: z =3.950, *p* = 0.000; *I*^2^ = 96.7%; PD-1 + target therapy vs. PD-1: z =3.211, *p* = 0.001; *I*^2^ =95.6%; PD-1 + radiotherapy vs. PD-1: z =2.171, *p* = 0.030; *I*^2^ =94.0%; PD-1 + chemotherapy vs. PD-1: z =2.564, *p* = 0.010; *I*^2^ = 98.6%; PD-1 + other vs. PD-1: z =0.324, *p* = 0.746; Overall: z =5.674, *p* = 0.000; *I*^2^ =97.5%; Between: *p* = 0.021.

**Table 4:** Subgroup analysis of any grade adverse events.

Experimental vs. Control	No. of Studies	RR	95% CI	*p*	Heterogeneity (I2)
Any grade adverse events	38	1.13	[1.08, 1.18]	<0.001	89%
Any grade fatigue	42	1.31	[1.15, 1.50]	<0.001	62%
Any grade diarrhea	37	1.87	[1.58, 2.21]	<0.001	59%
Any grade rash	37	1.81	[1.51, 2.16]	<0.001	64%
Any grade pruritus	34	1.48	[1.34, 1.64]	<0.001	39%
Any grade nausea	31	1.75	[1.32, 2.33]	<0.001	80%
Any grade thyroid abnormalities	31	1.22	[1.11, 1.35]	<0.001	26%
Any grade decreased appetite	30	1.59	[1.27, 2.00]	<0.001	69%
Any grade elevated enzymes	29	1.93	[1.61, 2.33]	<0.001	58%
Any grade hypothyroidism	27	1.2	[1.06, 1.35]	0.003	7%
Any grade pain	27	1.24	[1.03, 1.49]	0.02	56%
Any grade anemia	26	2.03	[1.54, 2.67]	<0.001	68%
Any grade pyrexia	25	1.89	[1.44, 2.48]	<0.001	55%
Any grade vomiting	22	2.34	[1.77, 3.08]	<0.001	56%
Any grade decreased white-cell count	20	2.56	[1.16, 5.67]	0.02	83%
Any grade decreased platelet count	19	2.83	[1.27, 6.29]	0.01	80%
Any grade constipation	18	1.79	[1.35, 2.38]	<0.001	63%
Any grade asthenia	15	1.51	[1.32, 1.72]	<0.001	23%
Any grade infection	11	1.65	[1.36, 2.01]	<0.001	12%
Any grade serious event	11	1.44	[1.21, 1.72]	<0.001	57%
Any grade treatment-related Adverse Events Leading to Discontinuation	11	1.83	[1.46, 2.29]	<0.001	0
Any grade treatment-related serious adverse events	10	2.35	[1.97, 2.81]	<0.001	11%

**Table 5:** Subgroup analysis of 3–5 grade adverse events.

Experimental vs. Control	No. of Studies	RR	95% CI	*p*	Heterogeneity(I2)
3–5 grade adverse events	39	1.81	[1.63, 2.01]	<0.001	77%
3–5 grade fatigue	41	1.93	[1.71, 2.18]	<0.001	39%
3–5 grade diarrhea	34	3.35	[2.46, 4.57]	<0.001	6%
3–5 grade rash	34	2.07	[1.45, 2.93]	<0.001	31%
3–5 grade pruritus	31	2	[0.89, 4.46]	=0.09	0%
3–5 grade elevated enzymes	30	2.05	[1.44, 2.92]	<0.001	55%
3–5 grade nausea	29	3.06	[2.02, 4.65]	<0.001	37%
3–5 grade decreased appetite	28	2.06	[1.35, 3.16]	<0.001	24%
3–5 grade thyroid abnormalities	27	1.82	[0.83, 3.97]	0.13	0%
3–5 grade anemia	25	4.51	[2.64, 7.70]	<0.001	60%
3–5 grade pain	25	0.97	[0.66, 1.43]	0.88	0%
3–5 grade hypothyroidism	24	1.13	[0.40, 3.18]	0.81	0%
3–5 grade decreased white-cell count	21	3.44	[1.06, 11.19]	0.04	54%
3–5 grade pyrexia	21	2.67	[1.42, 5.05]	0.002	0%
3–5 grade vomiting	20	3.91	[1.51, 10.07]	0.005	63%
3–5 grade decreased platelet count	19	6.12	[1.86, 20.16]	0.003	66%
3–5 grade asthenia	17	2.39	[1.68, 3.40]	<0.001	8%
3–5 grade constipation	17	0.82	[0.36, 1.90]	0.65	0%
3–5 grade infection	12	1.25	[1.00, 1.55]	0.05	35%
3–5 grade treatment-related Adverse Events Leading to Discontinuation	8	3.4	[2.26, 5.12]	<0.001	0%
3–5 grade treatment-related serious adverse events	6	2.84	[1.65, 4.86]	<0.001	0%
3–5 serious event	4	2.29	[1.48, 3.53]	<0.001	0%

## Data Availability

All data generated or analyzed during this study are included in this article.

## Supplementary Materials

The following are available online at https://www.mdpi.com/article/10.3390/cancers15030682/s1, Figure S1: Risk of bias graph: covers 45 studies on the risk of bias judgments, Table S1: Search strategy, Figure S2: Risk of bias summary: covers 45 studies on the risk of bias judgments, Table S2: Baseline demographics and clinical characteristics, Figure S3: Forest Plot of the Risk ratio of ORR based on tumor types: anti-PD-1/PD-L1 versus combination therapy (digestive system tumors, genitourinary system tumors, non-small cell lung cancer, head and neck squamous cell carcinoma, melanoma, glioblastoma, small cell lung cancer, malignant pleural mesothelioma, nasopharyngeal carcinoma, and classic Hodgkin’s lymphoma), Table S3: Median survival ratio of OS based on tumor types, Figure S4: Forest Plot of the Risk ratio of DCR based on tumor types: anti-PD-1/PD-L1 versus combination therapy (digestive system tumors, genitourinary system tumors, non-small cell lung cancer, head and neck squamous cell carcinoma, melanoma, glioblastoma, small cell lung cancer, malignant pleural mesothelioma, and nasopharyngeal carcinoma), Table S4: Median survival ratio of PFS based on tumor types, Figure S5: Forest Plot of the Hazard ratio of OS based on tumor types: anti-PD-1/PD-L1 versus combination therapy (genitourinary system tumors, non-small cell lung cancer, head and neck squamous cell carcinoma, and melanoma), Figure S6: Forest Plot of the Hazard ratio of PFS based on therapeutic schedules: anti-PD-1/PD-L1 versus combination therapy (immunotherapy, targeted therapy, radiotherapy, chemotherapy, and other drugs), Figure S7: Forest Plot of the Hazard ratio of PFS based on tumor types: anti-PD-1/PD-L1 versus combination therapy (digestive system tumors, genitourinary system tumors, non-small cell lung cancer, head and neck squamous cell carcinoma, and melanoma), Figure S8: Begg’s test (P=0.406), Egger’s test (P=0.931), and Harbord’s test (P=0.952) showed no significant publication bias in ORR, Figure S9: Begg’s test (P=0.448), Egger’s test (P=0.554), and Harbord’s test (P=0.348) showed no significant publication bias in DCR, Figure S10: Begg’s test and Egger’s test (A)Begg’s test (P=0.533) and Egger’s test (P=0.735) showed no significant publication bias in Hazard ratio of OS (B)Begg’s test (P=0.669) and Egger’s test (P=0.383) showed no significant publication bias in Hazard ratio of PFS, Figure S11: Begg’s test and Egger’s test (A)Begg’s test (P=0.803) and Egger’s test (P=0.860) showed no significant publication bias in median OS (B)Begg’s test (P=0.204) and Egger’s test (P=0.952) showed no significant publication bias in median PFS.
