# Supplementary material for: Efficacy and Safety of PD-1/PD-L1 Checkpoint Inhibitors versus Anti-PD-1/PD-L1 Combined with Other Therapies for Tumors: A Systematic Review
Source: Cancers (Basel). 2023 Jan 22;15(3):682. doi: 10.3390/cancers15030682 (PMC9913120; doi:10.3390/cancers15030682)
Supplement: Supplementary file 1 [file cancers-15-00682-s001.zip › cancers-2088343-supplementary.pdf]

Supplementary data

Efficacy and Safety of PD-1/PD-L1 Checkpoint Inhibitors versus Anti-PD-1/PD-L1 Combined with Other Therapies for Tumors: A Systematic Review

Table S1 Search strategy

#1 ("Programmed Cell Death 1 Receptor"[Mesh]) OR ((((((((((((PD-1 Protein[Title/Abstract]) OR (PD 1 Protein[Title/Abstract])) OR (PD-1 Receptor[Title/Abstract])) OR (PD 1 Receptor[Title/Abstract])) OR (Receptor, PD-1[Title/Abstract])) OR (Antigens, CD279[Title/Abstract])) OR (CD279 Antigens[Title/Abstract])) OR (CD279 Antigen[Title/Abstract])) OR (Antigen, CD279[Title/Abstract])) OR (PD1 Receptor[Title/Abstract])) OR (Receptor, PD1[Title/Abstract])) OR (Programmed Cell Death Protein 1[Title/Abstract])) OR (Programmed Cell Death 1 Protein[Title/Abstract]))

#2 ("Immune Checkpoint Inhibitors"[Mesh]) OR (((((((((((((((((((((((((((((((Checkpoint Inhibitors, Immune[Title/Abstract]) OR (Immune Checkpoint Inhibitor[Title/Abstract])) OR (Checkpoint Inhibitor, Immune[Title/Abstract])) OR (Immune Checkpoint Blockers[Title/Abstract])) OR (Checkpoint Blockers, Immune[Title/Abstract])) OR (Immune Checkpoint Blockade[Title/Abstract])) OR (Checkpoint Blockade, Immune[Title/Abstract])) OR (Immune Checkpoint Inhibition[Title/Abstract])) OR (Checkpoint Inhibition, Immune[Title/Abstract])) OR (PD-L1 Inhibitors[Title/Abstract])) OR (PD L1 Inhibitors[Title/Abstract])) OR (PD-L1 Inhibitor[Title/Abstract])) OR (PD L1 Inhibitor[Title/Abstract])) OR (Programmed Death-Ligand 1 Inhibitors[Title/Abstract])) OR (Programmed Death Ligand 1 Inhibitors[Title/Abstract])) OR (PD-1-PD-L1 Blockade[Title/Abstract])) OR (Blockade, PD-1-PD-L1[Title/Abstract])) OR (PD 1 PD L1 Blockade[Title/Abstract])) OR (CTLA-4 Inhibitors[Title/Abstract])) OR (CTLA 4 Inhibitors[Title/Abstract])) OR (CTLA-4 Inhibitor[Title/Abstract])) OR (CTLA 4 Inhibitor[Title/Abstract])) OR (Cytotoxic T-Lymphocyte-Associated Protein 4 Inhibitors[Title/Abstract])) OR (Cytotoxic T Lymphocyte Associated Protein 4 Inhibitors[Title/Abstract])) OR (Cytotoxic T-Lymphocyte-Associated Protein 4 Inhibitor[Title/Abstract])) OR (Cytotoxic T Lymphocyte Associated Protein 4 Inhibitor[Title/Abstract])) OR (PD-1 Inhibitors[Title/Abstract])) OR (PD 1 Inhibitors[Title/Abstract])) OR (PD-1 Inhibitor[Title/Abstract])) OR (Inhibitor, PD-1[Title/Abstract])) OR (PD 1

Inhibitor[Title/Abstract])) OR (Programmed Cell Death Protein 1 Inhibitor[Title/Abstract])) OR (Programmed Cell Death Protein 1 Inhibitors[Title/Abstract]))

#3 ((((((((Nivolumab[Title/Abstract]) OR (Pembrolizumab[Title/Abstract])) OR (Cemiplimab[Title/Abstract])) OR (Toripalimab[Title/Abstract])) OR (Cindilimab[Title/Abstract])) OR (Camrelizumab[Title/Abstract])) OR (Atezolizumab[Title/Abstract])) OR (Avelumab[Title/Abstract])) OR (Durvalumab[Title/Abstract])

#4 #1 OR #2 OR #3

#5 ("Neoplasms"[Mesh]) OR ((((((((((((((((Tumor[Title/Abstract]) OR (Neoplasm[Title/Abstract])) OR (Tumors[Title/Abstract])) OR (Neoplasia[Title/Abstract])) OR (Neoplasias[Title/Abstract])) OR (Cancer[Title/Abstract])) OR (Cancers[Title/Abstract])) OR (Malignant Neoplasm[Title/Abstract])) OR (Malignancy[Title/Abstract])) OR (Malignancies[Title/Abstract])) OR (Malignant Neoplasms[Title/Abstract])) OR (Neoplasm, Malignant[Title/Abstract])) OR (Neoplasms, Malignant[Title/Abstract])) OR (Benign Neoplasms[Title/Abstract])) OR (Benign Neoplasm[Title/Abstract])) OR (Neoplasms, Benign[Title/Abstract])) OR (Neoplasm, Benign[Title/Abstract]))

#6 #4 AND #5 filters: from 1000/1/1 - 2022/2/16

Table S2 Baseline demographics and clinical characteristics

Author	Study type	Region	Male : Female	Median follow-up, months		Median age (range), years		ECAEs	RECT
				experimental : control	total	experimental : control	total		
Boyer 2021[15]	Randomized, double-blind	24 countries	393 : 175	NR	20.6	64(35-85) : 65(35-85)	NR	NCI-CTCAE v.4.0	RECIST v.1.1
D'Angelo 2018[16]	Open-label, non-comparative, randomised	USA	41 : 44	14.2 : 13.6	NR	57.0 (27.0–81.0) : 56.0 (21.0–76.0)	NR	NCI-CTCAE v.4.0	RECIST v.1.1
Ferrarotto 2020[17]	Randomized	USA	28 : 1	NR	15.79	NR	NR	NCI-CTCAE v.4.03	RECIST v.1.1
Ferris 2020[18]	Randomized, open-label	NR	411 : 76	6.3 : 7.6	NR	61.0 (23–81) : 59.0 (24–84)	NR	NCI-CTCAE v.4.03	RECIST v.1.1
Gettinger 2021[19]	Randomized, multicenter, open-label	NR	169 : 83	NR	29.5	67.5 (41.8-83.4) : 68.1 (48.6-90.3)	67.5 (41.8-90.3)	NCI-CTCAE v.4.03	RECIST v.1.1
Janjigian 2018[20]	Open-label, two-stage, multicohort	USA and Europe	90 : 21	22 : 24	NR	58(19-81) : 60(29-80)	NR	NCI-CTCAE v.4.0	RECIST v.1.1

Kaseb 2022[21]	Single-centre, randomised, open-label	USA	19 : 8	NR	24.6	62 (53–72) : 64 (56–68)	64 (53–69)	NCI-CTCAE v.4.0	RECIST v.1.1
Kelley 2021[22]	Randomized, open-label	9 countries	157 : 22	NR	NR	66(26-86) : 64.5(32-89)	NR	NCI-CTCAE v.4.03	RECIST v.1.1
Kelly 2019[23]	Randomized, multicenter, open-label	6 countries	39 : 12	9.2 : 3.5	NR	64.0 (27–78) : 60.0 (29–79)	NR	NCI-CTCAE v.4.0	RECIST v.1.1
Long 2018[24]	Multicentre, open-label, randomised	Australia	48 : 12	14:17	17	59 (53–68) : 63 (52–74)	NR	NCI-CTCAE v.4.0	RECIST v.1.1
Long 2019[25]	International, randomised, placebo-controlled, double-blind, parallel-group	23 countries	423 : 283	NR	12.4	64 (52–72) : 63 (53.5–72)	64 (53–72)	NR	RECIST v.1.1
Omuro 2017[26]	Exploratory cohorts	USA	11 : 9	NR	NR	57 (37–68) : 58.5 (42–73)	NR	NCI-CTCAE v.4.0	RANO
O'Reilly 2019[27]	Multicenter, randomized, open-label	6 countries	34 : 31	NR	3.2	60.0 (37-81) : 62.0 (40-78)	61 (37-81)	NCI-CTCAE v.4.03	RECIST v.1.1

Paz-Ares 2021[27]	Randomized, open-label	North America, Europe, Asia and other 10 countries	527 : 265	NR	54.8	64 (26–84) : 64 (27–85)	NR	NR	NR
Planchard 2020[29]	Randomized, open-label, multicenter study	26 countries	188 : 103	NR	NR	62.5 (26–81) : 63.0 (19–83)	NR	NR	RECIST v.1.1
Powles 2020[30]	Open-label, multicentre, randomised, controlled	23 countries	505 : 183	NR	41.2	68 (60–73) : 67 (60–73)	NR	NCI-CTCAE v.4.03	RECIST v.1.1
Ready 2019[31]	Randomized, multicenter, open-label	8 countries	147 : 96	NR	NR	65.0 (41–91) : 63.0 (29–83)	NR	NCI-CTCAE v.4.0	RECIST v.1.1
Scherpereel 2019[32]	Multicentre, open-label, randomised, non-comparative	France	100 : 25	NR	20.1	71.2 (48.1–88.1) : 72.3 (32.5–87.2)	NR	NCI-CTCAE v.4.0	RECIST v.1.0
Schoenfeld 2020[33]	Open label, randomized	NR	18 : 11	NR	NR	65.2 (32.5-78.4) : 64.4 (39.1-81.0)	NR	NCI-CTCAE v.4.0	RECIST

Sharma 2019[34]	Multicenter, open-label, multiarm	8 countries	135 : 47	NR	NR	64(39-83) : 65.5(31-85)	NR	NCI-CTCAE v.4.0	RECIST v.1.1
Singh 2021[35]	Randomized , noncomparative, parallel group, unblinded	NR	19 : 17	NR	NR	NR	61 (21-80)	NCI-CTCAE v.4.03	RECIST v.1.1
Siu 2018[36]	Randomized, open-label, multicenter, global	North America, Europe and Asia Pacific	167 : 33	6.5 : 6.0	NR	62 (26-81) : 62 (23-82)	NR	NR	RECIST v.1.1
Tawbi 2022[37]	Global, double-blind, randomized	America, Europe, Australia and New Zealand	416 : 298	NR	13.2	63.0 (20–94) : 62.0 (21–90)	63 (20–94)	NCI-CTCAE v.5.0	RECIST v.1.1
Wolchok 2021[38]	Randomized	5 countries	408 : 222	57.5 : 36	NR	61 (18-88) : 60 (25-90)	NR	NCI-CTCAE v.4.0	RECIST v.1.1
Zamarin 2020[39]	Open-label, randomized	USA	all female	NR	NR	62(38-92) : 63(37-87)	NR	NCI-CTCAE v.4.0	RECIST v.1.1
Zimmer 2020[40]	Randomised, double-blind, placebo-controlled	Germany	62 : 53	NR	28.4	52 (45–59) : 57 (48–65)	NR	NCI-CTCAE v.4.0	RECIST v.1.1

Eng 2019[41]	Multicentre, open-label, randomised, controlled	11 countries	166 : 107	NR	7.3	58 (51–67) : 56 (51–64)	NR	NCI-CTCAE v.4.0	RECIST v.1.1
Gogas 2020[42]	International, randomized, open-label	North America, Europe and others	270 : 176	7.1 : 7.2	NR	66 (54-73) : 66 (55-73)	NR	NCI-CTCAE v.4.0	RECIST v.1.1
Lee 2020[43]	Open-label, multicentre, multiarm	7 countries	103 : 16	6.6 : 6.7	12.4	60 (22–82) : 63 (23–85)	NR	NCI-CTCAE v.4.0	RECIST v.1.1
McDermott 2018[44]	Randomised	NR	151 : 53	NR	20.7	62 (32-88) : 61 (27-81)	NR	NR	RECIST v.1.1
Nayak 2020[45]	Multicenter, open-label, two-cohort	NR	54 : 26	NR	NR	52 (42–59) : 55 (42–62)	53 (42–60)	NCI-CTCAE v.4.0	RANO
Taylor 2022[46]	Multicenter, open-label, randomized	USA	69 : 7	6.4 : 9.2	NR	58.0 (45.0–97.0) : 61.0 (38.0–83.0)	60.5 （38-97）	NCI-CTCAE v.4.03	RECIST v.1.1
Yarchoan 2021[47]	Randomised	USA	29 : 48	NR	NR	NR	NR	NR	RECIST v.1.1
Zhang 2020[48]	Randomized, open-label, multicenter	USA	57 : 18	NR	NR	68 (36-83) : 65 (46-96)	NR	NCI-CTCAE v.4.03	RECIST v.1.1

Altorki 2021[49]	Single-centre, open-label, randomised, controlled	USA	31 : 29	NR	16.9	70.0 (64.2–74.0) : 71.0 (65.2–75.0)	NR	NCI-CTCAE v.4.0	RECIST v.1.1.13
McBride 2020[50]	Single-center, randomized	USA	NR	NR	20.2	66(35-83) : 60.5(29-77)	63 (29-83)	NCI-CTCAE v.4.03	RECIST v.1.1
Papadopoulos 2019[51]	Open-label, multicenter	NR	NR	NR	4.4	NR	58（31-79）	NCI-CTCAE v.4.03	RECIST v.1.1
Theelen 2019[52]	Multicenter, randomized	Netherlands	44 : 32	NR	23.6	NR	62（35-78）	NCI-CTCAE v.4.0	RECIST v.1.1
Burtness 2019[53]	Randomised, open-label	37 countries	474 : 108	13.0 : 11.5	NR	61.0 (55.0–68.0) : 62.0 (56.0–68.0)	NR	NCI-CTCAE v.4.0	RECIST v.1.1
Fang 2018[54]	Two single-arm	China	92 : 24	10.2 : 9.9	NR	44 (34–51) : 45 (38–52)	NR	NCI-CTCAE v.4.03	RECIST v.1.1
Galsky 2020[55]	Multicentre, randomised, placebo-controlled	35 countries	618 : 195	NR	11.8	69 (62–75) : 67 (62–74)	NR	NCI-CTCAE v.4.0	RECIST v.1.1

Levy 2019[56]	Multicenter, international, randomised, placebo-controlled, double-blind	USA and Europe	57 : 43	11.3 : 12.2	NR	65 (39–82) : 66 (39–82)	65 (39–82)	NCI-CTCAE v.4.0	RECIST v.1.1
Nie 2019[57]	Two-arm, open-label	China	37 : 24	NR	14.9	26(18.3-31.8) : 28(23.5-32)	NR	NCI-CTCAE v.4.0	RRCML
Powles 2021[58]	Randomised, open-label	21 countries	500 : 158	NR	31.7	69 (62–75) : 68 (61–74)	NR	NCI-CTCAE v.4.0	RECIST v.1.1
Shitara 2020[59]	Randomized, controlled, partially blinded interventional	29 countries	375 : 138	NR	29.4	62.0 (22-83) : 61.0 (20-83)	NR	NCI-CTCAE v.4.0	RECIST v.1.1
Ueno 2019[60]	Non-randomised, multicentre, open-label	Janpan	32 : 28	8.2 : 5.1	NR	67.5 (54–75) : 68 (60–71)	NR	NCI-CTCAE v.4.0	RECIST v.1.1
Gutierrez 2020[61]	Open-label	7 countries	NE*	NR	NR	NR	55-69	NCI-CTCAE v.4.03	RECIST v.1.1
Spigel 2020[62]	Randomised	NR	60 : 41	NR	10	69 (49-82) : 68 (49-83)	NR	NCI-CTCAE v.4.03	RECIST v.1.1

NCI-CTCAE: National Cancer Institute Common Terminology Criteria for Adverse Events. RECIST: Response Evaluation Criteria In Solid Tumors. RANO: Response Assessment in Neuro-Oncology. RRCML: Revised Response Criteria for Malignant Lymphomas (2014 Lugano classification). NR: not reported. NE: not estimate; * The monotherapy group was described together with another group which we are not focused on in the baseline. ECAEs: Evaluation criteria of AEs. RECT: Response evaluation criteria in tumors.

Table S3 Median survival ratio of OS based on tumor types

subgroup and Author (Year)	ES	[95% Conf. Interval]		% Weight
NSCLC				
Planchard 2020[29]	1.150	1.025	1.290	3.55
Paz-Ares 2021[28]	1.089	1.016	1.168	3.66
Boyer 2021[15]	0.977	0.900	1.061	3.63
Gettinger 2021[19]	0.909	0.804	1.029	3.53
Theelen 2019[52]	2.092	1.671	2.620	3.16
Subgroup, DL	1.151	0.978	1.355	17.52
SCLC				
Ready 2019[31]	0.825	0.727	0.935	3.52
Subgroup, DL	0.825	0.727	0.935	3.52
HNSCC				
Burtness 2019[53]	0.872	0.804	0.946	3.63
Siu 2018[36]	1.267	1.100	1.458	3.48
Taylor 2022[46]	1.010	0.807	1.265	3.16
Ferris 2020[18]	0.855	0.783	0.935	3.62
McBride 2020[50]	0.979	0.760	1.261	3.03
Subgroup, DL	0.978	0.844	1.135	16.91
genitourinary system				
Zamarin 2020[39]	1.289	1.060	1.568	3.27
Galsky 2020[55]	1.019	0.951	1.092	3.66
Sharma 2019[34]	0.747	0.646	0.864	3.46
Powles 2020[30]	1.144	1.062	1.233	3.65
Powles 2021[58]	1.090	1.010	1.176	3.64
Zhang 2020[48]	0.553	0.441	0.693	3.15
Subgroup, DL	0.955	0.821	1.110	20.83
digestive system				

Singh 2021[35]	0.327	0.235	0.456	2.68
Eng 2019[41]	1.249	1.110	1.407	3.54
O'Reilly 2019[27]	0.861	0.675	1.098	3.08
Shitara 2020[59]	1.179	1.081	1.286	3.62
Ueno 2019[60]	2.962	2.299	3.814	3.03
Kelley 2021[22]	1.238	1.070	1.434	3.46
Kelly 2019[23]	2.706	2.056	3.560	2.94
Janjigian 2018[20]	0.774	0.643	0.932	3.31
Subgroup, DL	1.164	0.868	1.561	25.66
recurrent glioblastoma				
Omuro 2017[26]	0.885	0.564	1.387	2.17
Nayak 2020[45]	0.854	0.686	1.064	3.18
Subgroup, DL	0.860	0.706	1.047	5.35
relapsed malignant pleural mesothelioma				
Scherpereel 2019[32]	1.336	1.121	1.592	3.35
Subgroup, DL	1.336	1.121	1.592	3.35
melanoma				
Wolchok 2021[38]	1.954	1.807	2.113	3.64
Subgroup, DL	1.954	1.807	2.113	3.64
metastatic sarcoma				
D'Angelo 2018[16]	1.336	1.081	1.653	3.21
Subgroup, DL	1.336	1.081	1.653	3.21
Overall, DL	1.086	0.980	1.203	100.00

Tests of subgroup effect size = 1; Cochran's Q statistics for heterogeneity
NSCLC: z =1.688, P = 0.091, I^2 = 91.5%; SCLC: z =-3.007, P = 0.003; HNSCC: z =-0.288, P = 0.773, I^2 = 84.0%; Genitourinary system: z =-0.603, P = 0.546, I^2 = 92.0%; Digestive system: z =1.014, P = 0.311, I^2= 95.9%; Recurrent glioblastoma: z =-1.500, P = 0.134, I^2 = 0.0%; Relapsed malignant pleural me: z =3.240, P = 0.001; Melanoma: z =16.813, P = 0.000; Metastatic sarcoma: z =2.674, P = 0.007; Overall: z =1.568, P = 0.117, I^2 = 95.2%; Between: P = 0.000

Table S4 Median survival ratio of PFS based on tumor types

subgroup and Author (Year)	ES	[95% Conf. Interval]		% Weight
NSCLC				
Levy 2019[56]	0.725	0.596	0.882	2.70
Planchard 2020[29]	1.129	1.006	1.266	2.80
Spigel 2020[62]	1.033	0.850	1.255	2.70
Paz-Ares 2021[28]	1.214	1.133	1.302	2.84
Boyer 2021[15]	0.976	0.899	1.060	2.83
Gettinger 2021[19]	1.310	1.158	1.483	2.80
Theelen 2019[52]	3.474	2.774	4.349	2.66
Subgroup, DL	1.231	0.992	1.527	19.34
SCLC				
Ready 2019[31]	1.071	0.945	1.215	2.79
Subgroup, DL	1.071	0.945	1.215	2.79
HNSCC				
Burtness 2019[53]	2.130	1.964	2.311	2.83
Siu 2018[36]	1.053	0.914	1.212	2.78
Taylor 2022[46]	1.588	1.268	1.989	2.66
McBride 2020[50]	1.368	1.063	1.762	2.61
Subgroup, DL	1.490	1.006	2.205	10.87
genitourinary system				
Zamarin 2020[39]	1.950	1.603	2.372	2.70
McDermott 2018[44]	1.918	1.672	2.200	2.78
Sharma 2019[34]	0.929	0.803	1.074	2.77
Powles 2020[30]	1.609	1.493	1.734	2.84
Zhang 2020[48]	1.375	1.097	1.724	2.65
Subgroup, DL	1.503	1.169	1.933	13.75
digestive system				

Kaseb 2022[21]	2.078	1.425	3.030	2.35
Singh 2021[35]	0.710	0.510	0.989	2.45
Eng 2019[41]	0.985	0.874	1.109	2.80
O'Reilly 2019[27]	1.000	0.784	1.275	2.62
Shitara 2020[59]	3.450	3.164	3.762	2.83
Ueno 2019[60]	3.000	2.329	3.864	2.61
Yarchoan 2021[47]	1.952	1.533	2.484	2.63
Lee 2020[43]	1.647	1.376	1.971	2.73
Kelley 2021[22]	1.048	0.905	1.214	2.77
Kelly 2019[23]	1.125	0.855	1.480	2.56
Janjigian 2018[20]	1.143	0.949	1.377	2.72
Subgroup, DL melanoma	1.464	1.011	2.119	29.06
Long 2019[25]	0.959	0.891	1.033	2.84
Long 2018[24]	5.308	4.121	6.836	2.61
Gogas 2020[42]	0.965	0.876	1.063	2.82
Tawbi 2022[37]	2.196	2.040	2.363	2.84
Wolchok 2021[38]	1.667	1.541	1.802	2.84
Subgroup, DL recurrent glioblastoma	1.758	1.158	2.669	13.94
Omuro 2017[26]	0.789	0.504	1.238	2.18
Nayak 2020[45]	2.867	2.303	3.570	2.66
Subgroup, DL relapsed malignant pleural mesothelioma	1.528	0.432	5.406	4.85
Scherpereel 2019[32]	1.400	1.175	1.668	2.73
Subgroup, DL metastatic sarcoma	1.400	1.175	1.668	2.73

D'Angelo 2018[16]	2.412	1.950	2.983	2.68
Subgroup, DL	2.412	1.950	2.983	2.68
Overall, DL	1.473	1.285	1.688	100.00

Tests of subgroup effect size = 1; Cochran's Q statistics for heterogeneity

NSCLC: z =1.887, P = 0.059, I^2 = 95.6%; SCLC: z =1.075, P = 0.282; HNSCC: z =1.992, P = 0.046, I^2 = 96.1%; Genitourinary system: z =3.176, P = 0.001, I^2 = 93.8%; Digestive system: z =2.018, P = 0.044, I^2 = 97.9%; Melanoma: z =2.650, P = 0.008, I^2 = 99.1%; Recurrent glioblastoma: z =0.658, P = 0.511, I^2 = 96.1%; Relapsed malignant pleural me: z =3.762, P = 0.000; Metastatic sarcoma: z =8.117, P = 0.000; Overall: z =5.564, P = 0.000, I^2 = 97.5%; Between: P = 0.000

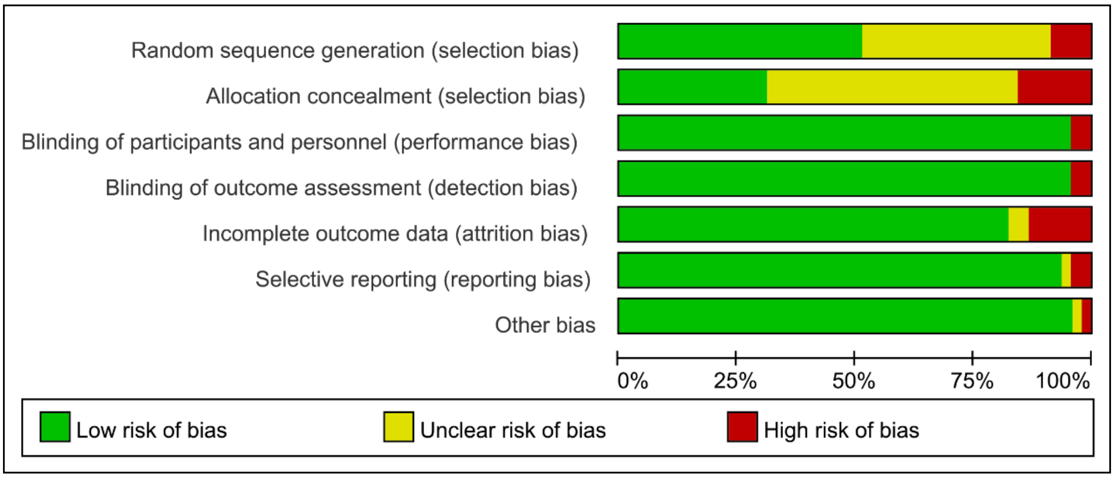

Figure S1 Risk of bias graph: covers 45 studies on the risk of bias judgments.

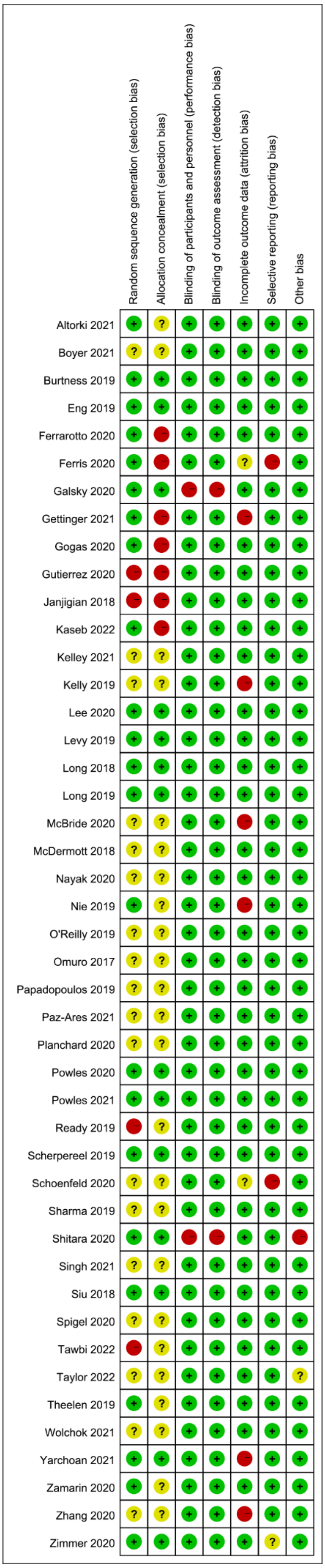

Figure S2 Risk of bias summary: covers 45 studies on the risk of bias judgments.

Study or Subgroup	Experimental Events	Experimental Total	Control Events	Control Total	Weight	Risk Ratio M-H, Random, 95% CI
**1.1.1 Digestive system**						
Eng 2019	5	183	2	90	0.6%	1.23 [0.24, 6.21]
Janjigian 2018	2	52	4	59	0.6%	0.57 [0.11, 2.97]
Kelley 2021	18	75	11	104	2.1%	2.27 [1.14, 4.52]
Kelly 2019	2	27	0	24	0.2%	4.46 [0.22, 88.61]
Lee 2020	16	60	10	59	2.1%	1.57 [0.78, 3.18]
O'Reilly 2019	1	32	0	33	0.2%	3.09 [0.13, 73.19]
Shitara 2020	125	257	38	256	3.9%	3.28 [2.38, 4.51]
Singh 2021	1	16	0	19	0.2%	3.53 [0.15, 81.11]
Ueno 2019	11	30	1	30	0.4%	11.00 [1.51, 79.96]
Yarchoan 2021	1	30	1	36	0.2%	1.20 [0.08, 18.38]
**Subtotal (95% CI)**		**762**		**710**	**10.4%**	**2.41 [1.68, 3.46]**
Total events	182		67			
Heterogeneity: Tau^2 = 0.05; Chi^2 = 10.67, df = 9 (P = 0.30); I^2 = 16%						
Test for overall effect: Z = 4.78 (P < 0.00001)						
**1.1.2 Genitourinary system**						
Galsky 2020	212	447	82	359	4.5%	2.08 [1.68, 2.57]
Gutierrez 2020	0	6	0	2		Not estimable
McDermott 2018	32	101	26	103	3.2%	1.26 [0.81, 1.95]
Powles 2020	124	342	89	346	4.4%	1.41 [1.12, 1.77]
Powles 2021	192	351	93	307	4.5%	1.81 [1.49, 2.19]
Sharma 2019	28	104	20	78	3.0%	1.05 [0.64, 1.72]
Zamarin 2020	16	51	6	49	1.6%	2.56 [1.09, 6.01]
Zhang 2020	8	40	9	35	1.7%	0.78 [0.34, 1.80]
**Subtotal (95% CI)**		**1442**		**1279**	**22.9%**	**1.54 [1.25, 1.91]**
Total events	612		325			
Heterogeneity: Tau^2 = 0.04; Chi^2 = 15.94, df = 6 (P = 0.01); I^2 = 62%						
Test for overall effect: Z = 3.97 (P < 0.0001)						
**1.1.3 NSCLC**						
Altorki 2021	29	30	25	30	4.6%	1.16 [0.98, 1.38]
Boyer 2021	129	284	129	284	4.6%	1.00 [0.84, 1.20]
Gettinger 2021	23	125	22	127	2.8%	1.06 [0.63, 1.80]
Levy 2019	10	51	7	49	1.6%	1.37 [0.57, 3.32]
Paz-Ares 2021	144	396	109	396	4.5%	1.32 [1.07, 1.62]
Planchard 2020	26	174	18	117	2.7%	0.97 [0.56, 1.69]
Spigel 2020	24	51	22	50	3.3%	1.07 [0.70, 1.64]
Theelen 2019	17	36	9	40	2.2%	2.10 [1.07, 4.11]
**Subtotal (95% CI)**		**1147**		**1093**	**26.2%**	**1.15 [1.03, 1.28]**
Total events	402		341			
Heterogeneity: Tau^2 = 0.00; Chi^2 = 7.88, df = 7 (P = 0.34); I^2 = 11%						
Test for overall effect: Z = 2.45 (P = 0.01)						
**1.1.4 HNSCC**						
Burtness 2019	100	281	51	301	4.0%	2.10 [1.56, 2.82]
Ferris 2020	45	247	43	240	3.6%	1.02 [0.70, 1.48]
McBride 2020	9	31	10	29	1.9%	0.84 [0.40, 1.77]
Siu 2018	10	129	6	65	1.4%	0.84 [0.32, 2.21]
Taylor 2022	5	37	7	39	1.2%	0.75 [0.26, 2.16]
**Subtotal (95% CI)**		**725**		**674**	**12.1%**	**1.13 [0.70, 1.84]**
Total events	169		117			
Heterogeneity: Tau^2 = 0.19; Chi^2 = 14.15, df = 4 (P = 0.007); I^2 = 72%						
Test for overall effect: Z = 0.50 (P = 0.61)						
**1.1.5 Melanoma**						
Gogas 2020	53	204	65	206	4.0%	0.82 [0.61, 1.12]
Long 2018	16	35	5	25	1.6%	2.29 [0.96, 5.42]
Long 2019	121	354	111	352	4.5%	1.08 [0.88, 1.34]
Wolchok 2021	183	314	142	316	4.7%	1.30 [1.11, 1.51]
**Subtotal (95% CI)**		**907**		**899**	**14.7%**	**1.13 [0.89, 1.45]**
Total events	373		323			
Heterogeneity: Tau^2 = 0.04; Chi^2 = 9.67, df = 3 (P = 0.02); I^2 = 69%						
Test for overall effect: Z = 1.00 (P = 0.32)						
**1.1.6 Glioblastoma**						
Nayak 2020	10	50	0	30	0.2%	12.76 [0.77, 210.27]
Omuro 2017	0	10	1	9	0.2%	0.30 [0.01, 6.62]
**Subtotal (95% CI)**		**60**		**39**	**0.4%**	**2.08 [0.05, 91.75]**
Total events	10		1			
Heterogeneity: Tau^2 = 5.22; Chi^2 = 3.31, df = 1 (P = 0.07); I^2 = 70%						
Test for overall effect: Z = 0.38 (P = 0.71)						
**1.1.7 SCLC**						
Ready 2019	21	96	17	147	2.5%	1.89 [1.05, 3.40]
**Subtotal (95% CI)**		**96**		**147**	**2.5%**	**1.89 [1.05, 3.40]**
Total events	21		17			
Heterogeneity: Not applicable						
Test for overall effect: Z = 2.13 (P = 0.03)						
**1.1.8 MPM**						
Scherpereel 2019	16	62	11	63	2.1%	1.48 [0.75, 2.93]
**Subtotal (95% CI)**		**62**		**63**	**2.1%**	**1.48 [0.75, 2.93]**
Total events	16		11			
Heterogeneity: Not applicable						
Test for overall effect: Z = 1.12 (P = 0.26)						
**1.1.9 NPC**						
Fang 2018	20	22	31	91	3.9%	2.67 [1.95, 3.66]
**Subtotal (95% CI)**		**22**		**91**	**3.9%**	**2.67 [1.95, 3.66]**
Total events	20		31			
Heterogeneity: Not applicable						
Test for overall effect: Z = 6.11 (P < 0.00001)						
**1.1.10 cHL**						
Nie 2019	40	42	17	19	4.7%	1.06 [0.90, 1.26]
**Subtotal (95% CI)**		**42**		**19**	**4.7%**	**1.06 [0.90, 1.26]**
Total events	40		17			
Heterogeneity: Not applicable						
Test for overall effect: Z = 0.73 (P = 0.47)						
**Total (95% CI)**		**5265**		**5014**	**100.0%**	**1.40 [1.23, 1.60]**
Total events	1845		1250			
Heterogeneity: Tau^2 = 0.09; Chi^2 = 151.60, df = 39 (P < 0.00001); I^2 = 74%						
Test for overall effect: Z = 5.01 (P < 0.00001)						
Test for subgroup differences: Chi^2 = 46.97, df = 9 (P < 0.00001), I^2 = 80.8%						

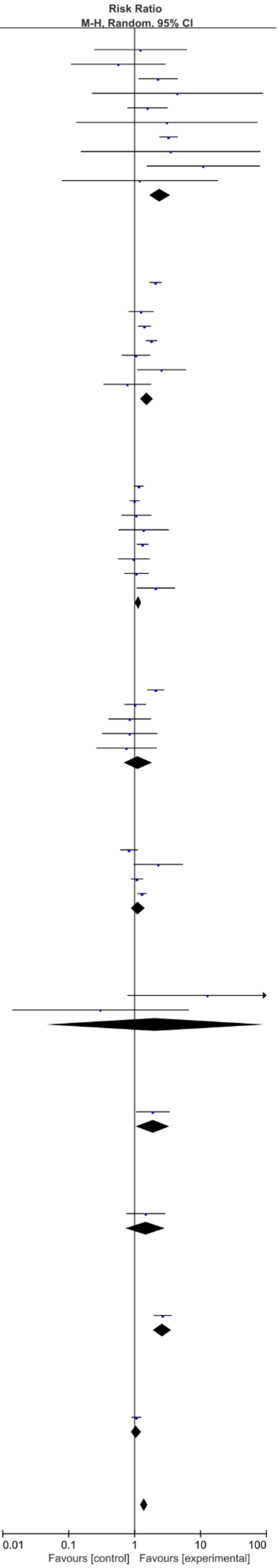

Figure S3 Forest Plot of the Risk ratio of ORR based on tumor types: anti-PD-1/PD-L1 versus combination therapy (digestive system tumors, genitourinary system tumors, non-small cell lung cancer, head and neck squamous cell carcinoma, melanoma, glioblastoma, small cell lung cancer, malignant pleural mesothelioma, nasopharyngeal carcinoma, and classic Hodgkin's lymphoma)

Study or Subgroup	Experimental Events	Experimental Total	Control Events	Control Total	Weight	Risk Ratio M-H, Random, 95% CI
**1.1.1 Digestive system**						
Eng 2019	48	183	19	90	2.3%	1.24 [0.78, 1.98]
Janjigian 2018	19	52	22	59	2.2%	0.98 [0.60, 1.60]
Kelley 2021	34	75	39	104	3.1%	1.21 [0.85, 1.72]
Kelly 2019	7	27	3	24	0.5%	2.07 [0.60, 7.14]
Lee 2020	41	60	29	59	3.5%	1.39 [1.02, 1.90]
O'Reilly 2019	3	32	2	33	0.3%	1.55 [0.28, 8.65]
Singh 2021	5	16	10	19	1.0%	0.59 [0.26, 1.38]
Ueno 2019	19	30	7	30	1.3%	2.71 [1.34, 5.48]
Yarchoan 2021	14	30	11	36	1.6%	1.53 [0.82, 2.85]
**Subtotal (95% CI)**		**505**		**454**	**15.9%**	**1.30 [1.06, 1.59]**
Total events	190		142			
Heterogeneity: Tau² = 0.02; Chi² = 10.04, df = 8 (P = 0.26); I² = 20%						
Test for overall effect: Z = 2.48 (P = 0.01)						
**1.1.2 Genitourinary system**						
Galsky 2020	344	447	173	359	5.2%	1.60 [1.42, 1.80]
Gutierrez 2020	0	6	2	2	0.1%	0.09 [0.01, 1.30]
Powles 2020	190	342	159	346	4.9%	1.21 [1.04, 1.40]
Powles 2021	282	351	145	307	5.1%	1.70 [1.50, 1.94]
Sharma 2019	52	104	41	78	3.7%	0.95 [0.72, 1.27]
Zamarin 2020	36	51	20	49	2.9%	1.73 [1.18, 2.53]
Zhang 2020	19	40	16	35	2.2%	1.04 [0.64, 1.69]
**Subtotal (95% CI)**		**1341**		**1176**	**24.2%**	**1.35 [1.11, 1.64]**
Total events	923		556			
Heterogeneity: Tau² = 0.04; Chi² = 29.37, df = 6 (P < 0.0001); I² = 80%						
Test for overall effect: Z = 3.05 (P = 0.002)						
**1.1.3 NSCLC**						
Altorki 2021	6	30	5	30	0.7%	1.20 [0.41, 3.51]
Boyer 2021	199	284	202	284	5.3%	0.99 [0.89, 1.10]
Levy 2019	13	51	19	49	1.8%	0.66 [0.37, 1.18]
Paz-Ares 2021	258	396	239	396	5.3%	1.08 [0.97, 1.20]
Spigel 2020	32	51	31	50	3.5%	1.01 [0.75, 1.37]
Theelen 2019	26	36	19	40	2.9%	1.52 [1.04, 2.23]
**Subtotal (95% CI)**		**848**		**849**	**19.4%**	**1.04 [0.93, 1.17]**
Total events	534		515			
Heterogeneity: Tau² = 0.01; Chi² = 7.67, df = 5 (P = 0.18); I² = 35%						
Test for overall effect: Z = 0.75 (P = 0.45)						
**1.1.4 HNSCC**						
Burtness 2019	178	281	133	301	4.9%	1.43 [1.23, 1.67]
Ferris 2020	102	247	99	240	4.4%	1.00 [0.81, 1.24]
Siu 2018	17	129	14	65	1.5%	0.61 [0.32, 1.16]
Taylor 2022	20	37	19	39	2.5%	1.11 [0.72, 1.72]
**Subtotal (95% CI)**		**694**		**645**	**13.3%**	**1.08 [0.80, 1.45]**
Total events	317		265			
Heterogeneity: Tau² = 0.06; Chi² = 12.33, df = 3 (P = 0.006); I² = 76%						
Test for overall effect: Z = 0.51 (P = 0.61)						
**1.1.5 Melanoma**						
Gogas 2020	126	204	118	206	4.8%	1.08 [0.92, 1.27]
Long 2018	20	35	5	25	1.0%	2.86 [1.24, 6.58]
Long 2019	180	354	179	352	5.0%	1.00 [0.86, 1.16]
Wolchok 2021	221	314	171	316	5.1%	1.30 [1.15, 1.47]
**Subtotal (95% CI)**		**907**		**899**	**16.0%**	**1.17 [0.97, 1.42]**
Total events	547		473			
Heterogeneity: Tau² = 0.02; Chi² = 12.53, df = 3 (P = 0.006); I² = 76%						
Test for overall effect: Z = 1.63 (P = 0.10)						
**1.1.6 Glioblastoma**						
Omuro 2017	3	10	5	9	0.6%	0.54 [0.18, 1.64]
**Subtotal (95% CI)**		**10**		**9**	**0.6%**	**0.54 [0.18, 1.64]**
Total events	3		5			
Heterogeneity: Not applicable						
Test for overall effect: Z = 1.09 (P = 0.28)						
**1.1.7 SCLC**						
Ready 2019	37	96	42	147	3.1%	1.35 [0.94, 1.93]
**Subtotal (95% CI)**		**96**		**147**	**3.1%**	**1.35 [0.94, 1.93]**
Total events	37		42			
Heterogeneity: Not applicable						
Test for overall effect: Z = 1.63 (P = 0.10)						
**1.1.8 MPM**						
Scherpereel 2019	32	62	25	63	2.9%	1.30 [0.88, 1.92]
**Subtotal (95% CI)**		**62**		**63**	**2.9%**	**1.30 [0.88, 1.92]**
Total events	32		25			
Heterogeneity: Not applicable						
Test for overall effect: Z = 1.33 (P = 0.18)						
**1.1.9 NPC**						
Fang 2018	22	22	54	91	4.7%	1.65 [1.38, 1.98]
**Subtotal (95% CI)**		**22**		**91**	**4.7%**	**1.65 [1.38, 1.98]**
Total events	22		54			
Heterogeneity: Not applicable						
Test for overall effect: Z = 5.46 (P < 0.00001)						
**Total (95% CI)**		**4485**		**4333**	**100.0%**	**1.22 [1.11, 1.34]**
Total events	2605		2077			
Heterogeneity: Tau² = 0.04; Chi² = 133.20, df = 33 (P < 0.00001); I² = 75%						
Test for overall effect: Z = 4.22 (P < 0.0001)						
Test for subgroup differences: Chi² = 22.61, df = 8 (P = 0.004), I² = 64.6%						

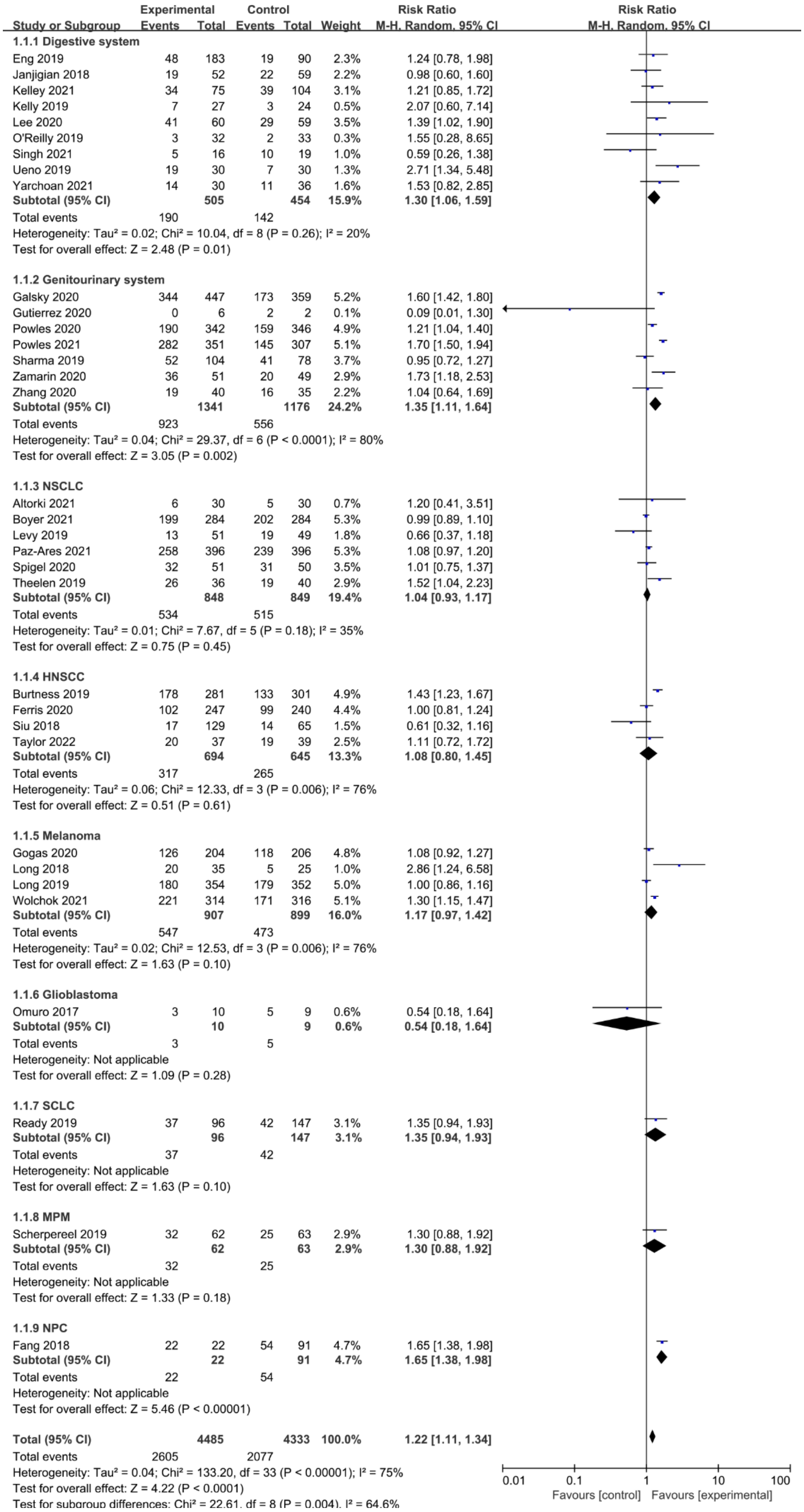

Figure S4 Forest Plot of the Risk ratio of DCR based on tumor types: anti-PD-1/PD-L1 versus combination therapy (digestive system tumors, genitourinary system tumors, non-small cell lung cancer, head and neck squamous cell carcinoma, melanoma, glioblastoma, small cell lung cancer, malignant pleural mesothelioma, and nasopharyngeal carcinoma)

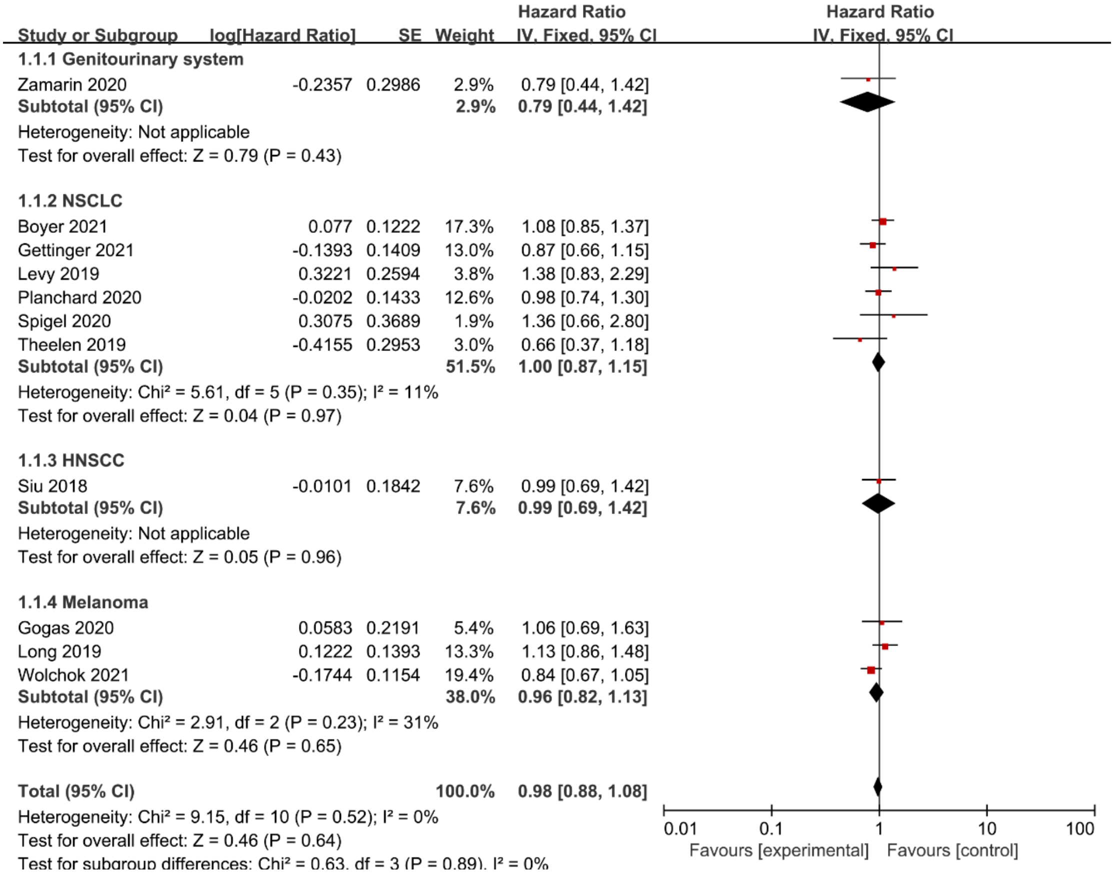

Figure S5 Forest Plot of the Hazard ratio of OS based on tumor types: anti-PD-1/PD-L1 versus combination therapy (genitourinary system tumors, non-small cell lung cancer, head and neck squamous cell carcinoma, and melanoma)

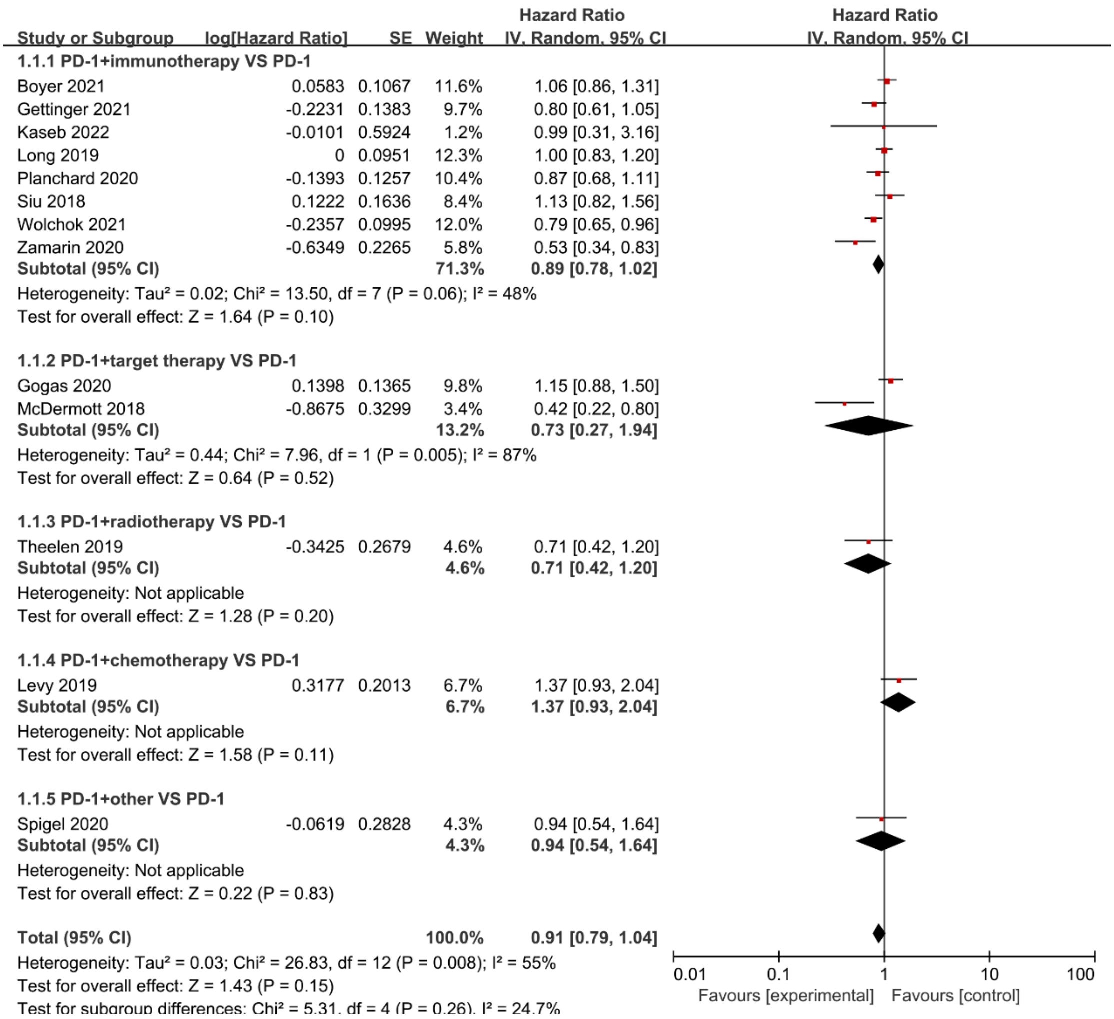

Figure S6 Forest Plot of the Hazard ratio of PFS based on therapeutic schedules: anti-PD-1/PD-L1 versus combination therapy (immunotherapy, targeted therapy, radiotherapy, chemotherapy, and other drugs)

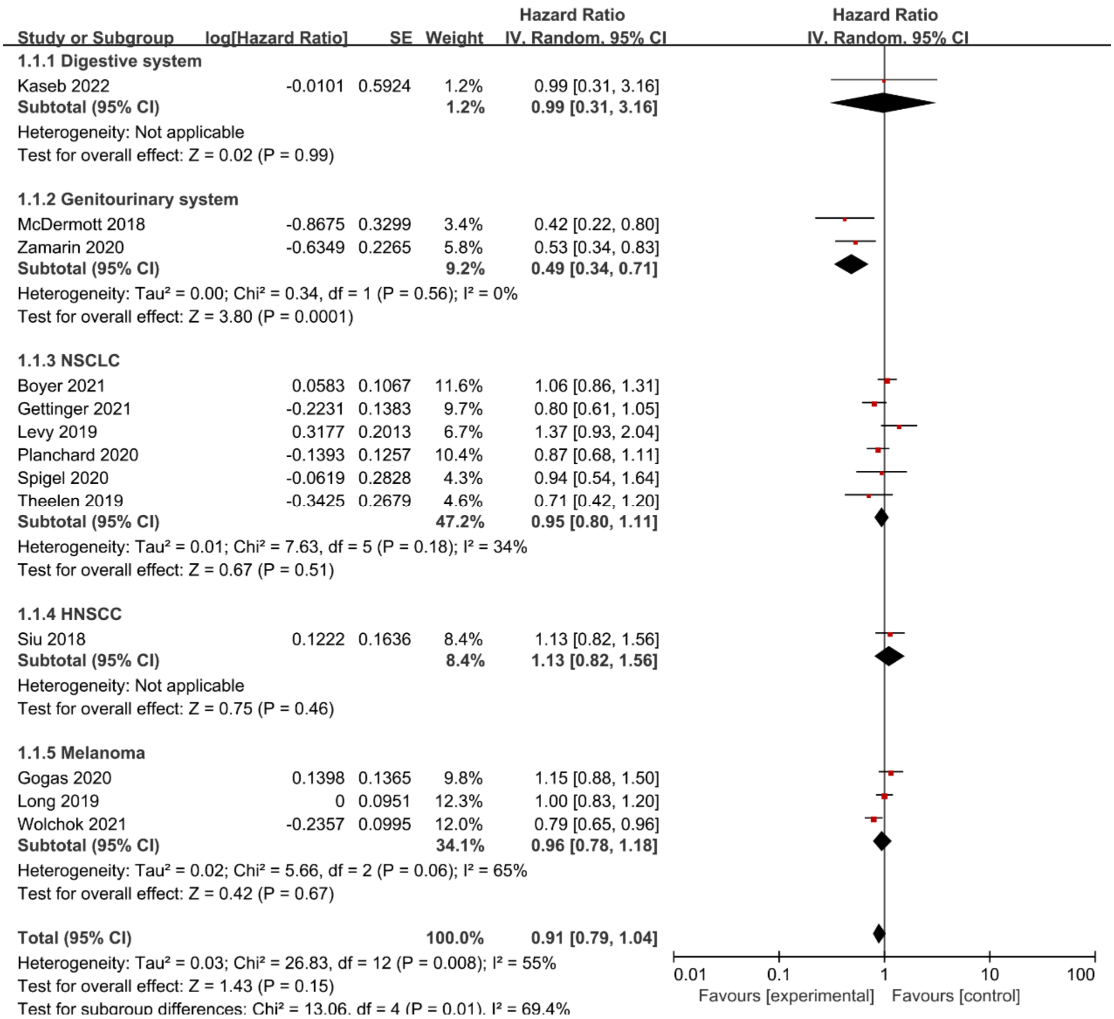

Figure S7 Forest Plot of the Hazard ratio of PFS based on tumor types: anti-PD-1/PD-L1 versus combination therapy (digestive system tumors, genitourinary system tumors, non-small cell lung cancer, head and neck squamous cell carcinoma, and melanoma)

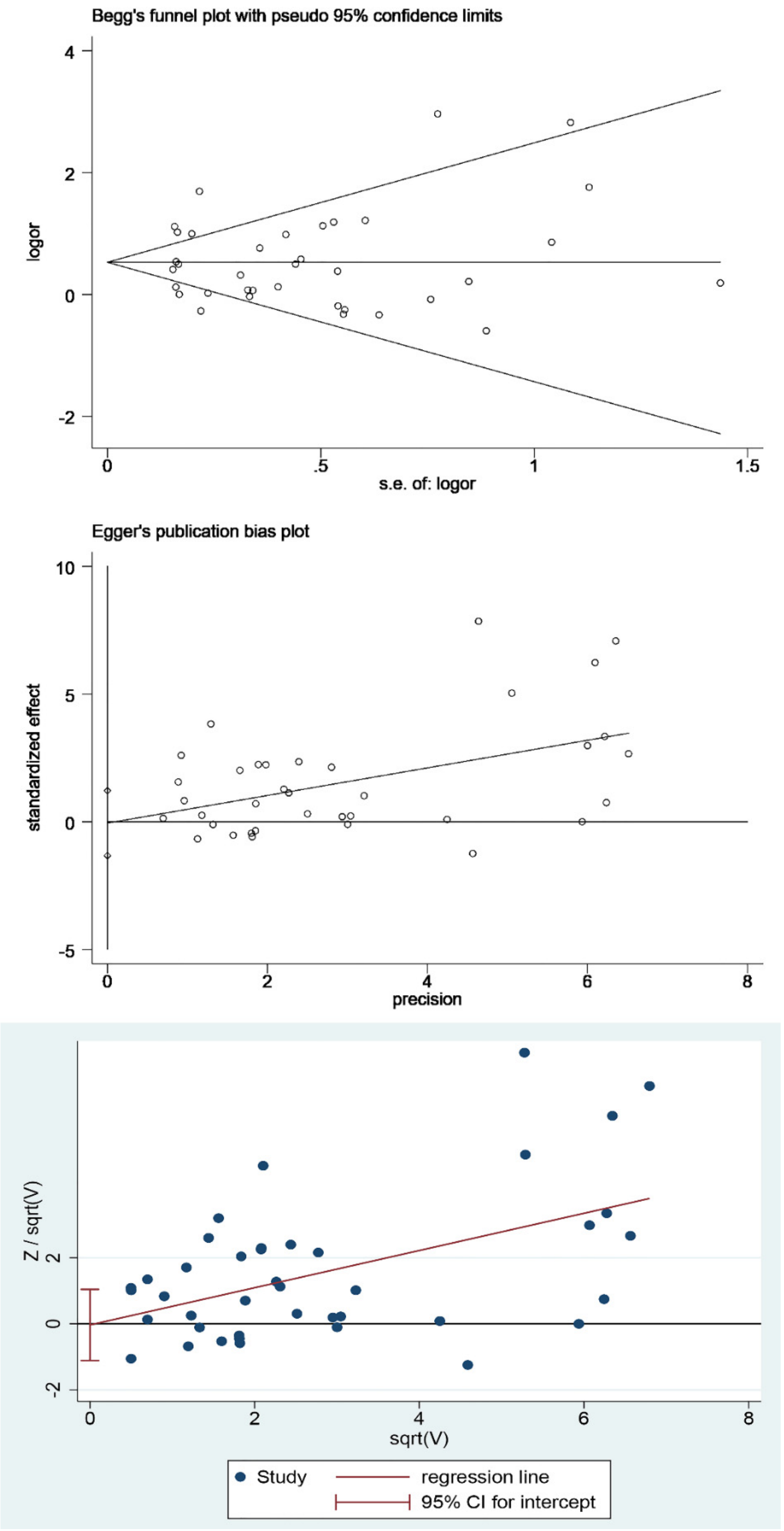

Figure S8 Begg's test (*P*=0.406), Egger's test (*P*=0.931), and Harbord's test (*P*=0.952) showed no significant publication bias in ORR.

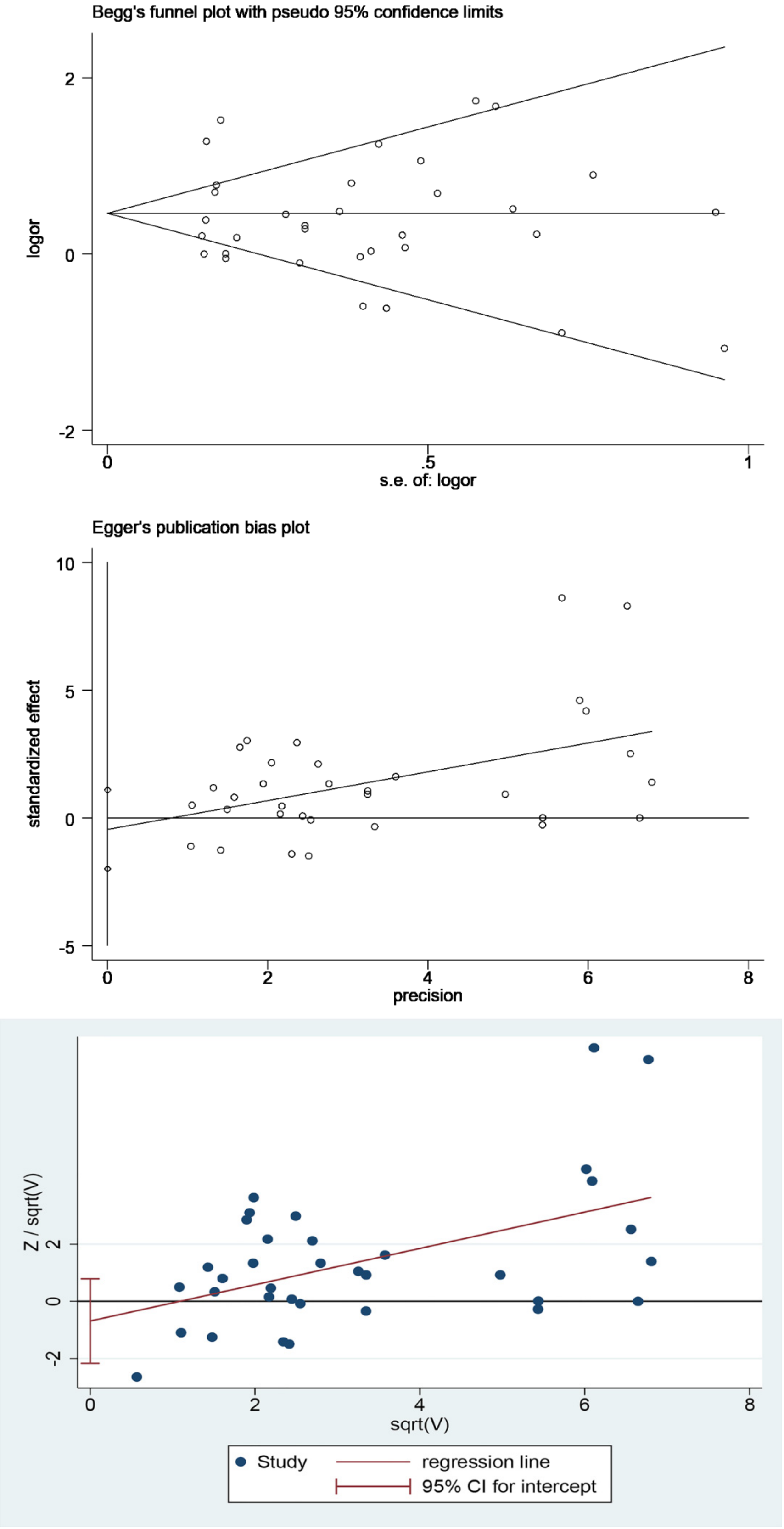

Figure S9 Begg's test (*P*=0.448), Egger's test (*P*=0.554), and Harbord's test (*P*=0.348) showed no significant publication bias in DCR.

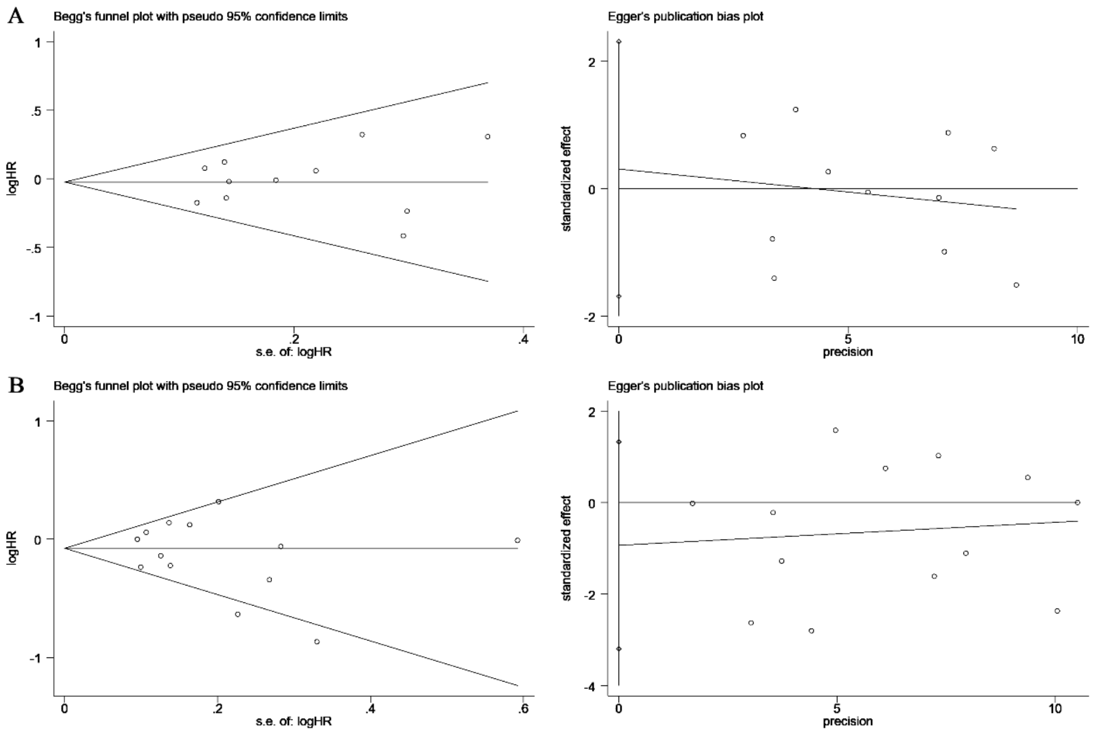

Figure S10 Begg's test and Egger's test

(A) Begg's test (*P*=0.533) and Egger's test (*P*=0.735) showed no significant publication bias in Hazard ratio of OS.

(B) Begg's test (*P*=0.669) and Egger's test (*P*=0.383) showed no significant publication bias in Hazard ratio of PFS.

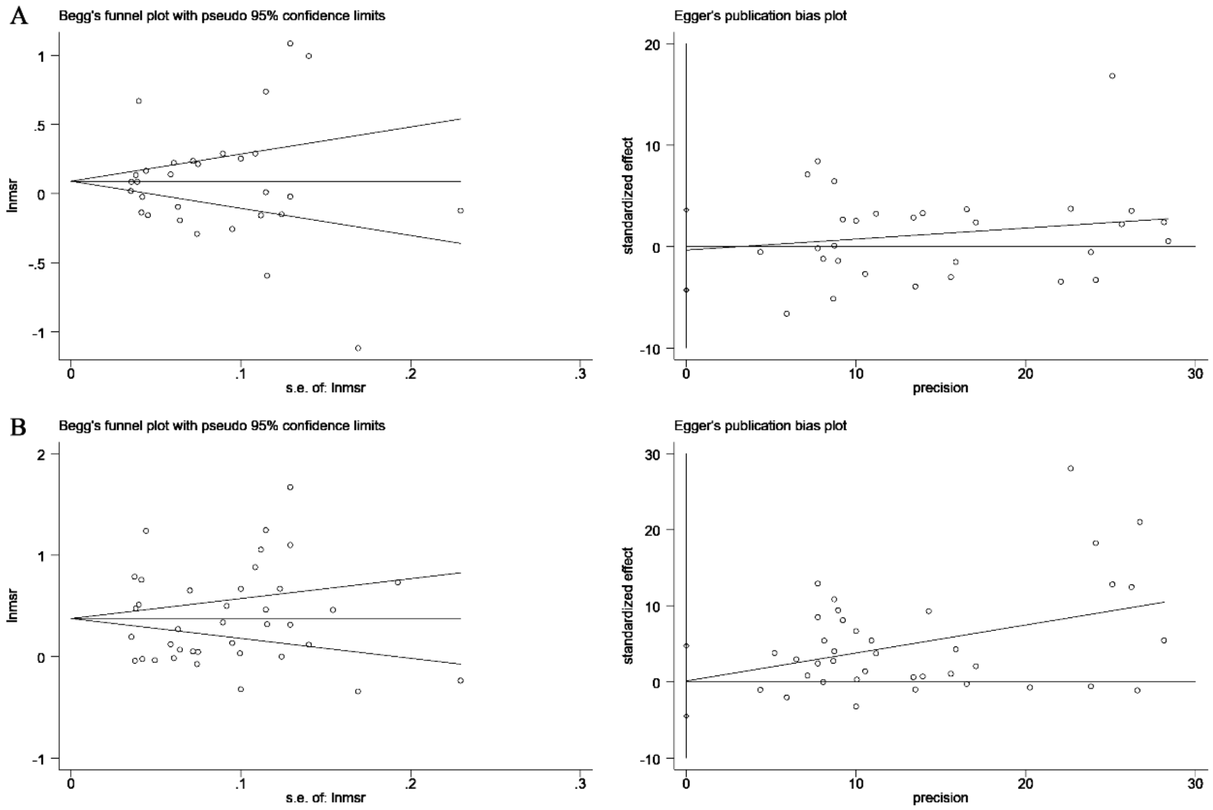

Figure S11 Begg's test and Egger's test

(A) Begg's test (*P*=0.803) and Egger's test (*P*=0.860) showed no significant publication bias in median OS.

(B) Begg's test (*P*=0.204) and Egger's test (*P*=0.952) showed no significant publication bias in median PFS.
